# Assessment of antigen-specific T cell recall responses in non-human primates using a composite AIM assay

**DOI:** 10.3389/fimmu.2025.1661480

**Published:** 2025-10-24

**Authors:** Jan M. Schmidt, Mayuri Prasad, Kenna Degner, Rebecca Schiavo, Allison Repic, Lauren Little, Laura Israel, Feng Chen, Regine Hansen, Anette C. Karle, Kees Leenhouts, Poul Sørensen, Brian McIntosh, Dominique Brees, José M. Carballido

**Affiliations:** ^1^ Novartis Biomedical Research, Preclinical Safety, Basel, Switzerland; ^2^ LabCorp, Early Development Laboratories, Madison, WI, United States; ^3^ Novartis Biomedical Research, Biologics Research Center, Basel, Switzerland; ^4^ Novartis Biomedical Research, DAx, Cambridge MA, United States; ^5^ Allero Therapeutics, Rotterdam, Netherlands; ^6^ Department of Biomedicine, Aarhus University, Aarhus, Denmark; ^7^ Department of Pharmaceuticals Sciences, University of Basel, Basel, Switzerland

**Keywords:** immunogenicity assessments, activation-induced marker assay, antigen-specific T cell recall responses, non-human primates, fluorescent and mass cytometry, biotherapeutic development

## Abstract

**Introduction:**

Characterizing antigen-specific T cell responses is essential for understanding the immunogenicity of biotherapeutics and mitigating drug-specific immune reactions.

**Methods:**

This study describes a flow cytometry composite Activation-Induced Marker (cAIM) assay for cynomolgus monkeys that allows quantification of T cell recall responses to multiple antigens using up to ten AIM pairs. The procedure incorporates two composite metrics (cAIM-index and cAIM-score) that facilitate the summation of T cell recall responses into interpretable numeric values, reducing reliance on multiple graphical comparisons. The assay is compatible with human and mouse samples and can utilize peripheral blood mononuclear cells or whole blood. Additionally, the method is well suited for the mass cytometry platform, enabling the detection of antigen-specific CD4^+^ and CD8^+^ T cell recall responses while providing deep immunophenotype information and consuming minimal blood sample volumes.

**Results:**

The assay successfully enables quantification of antigen-specific T cell recall responses across multiple antigens and species, while composite metrics streamline interpretation.

**Discussion:**

These protocols shall support preclinical and clinical immunogenicity assessments, advancing biotherapeutic development.

## Introduction

Biotherapeutics, a class of drugs including proteins, nucleic acids and cells have revolutionized the pharmaceutical field (1). Among those, monoclonal antibodies account for most approved biotherapeutics and have a remarkable impact on patient health, particularly in autoimmunity and cancer (2, 3). Adeno Associated Virus- (AAV)-based therapeutics represent another drug family with transformative clinical benefits. The efficacy of all these products is linked to their high specificity. However, this advantage may be offset by their potential to trigger immunogenic reactions, which can undermine therapeutic effectiveness, and pose safety concerns (4–6).

Immunogenicity assessments traditionally focus on humoral responses, particularly on the detection of Anti-Drug Antibodies (ADAs) (7). ADAs reflect the extent and type of immune reaction to the biotherapeutic, and high titers can reduce the efficacy of the drug and/or trigger adverse reactions (4, 5, 8), which can lead to discontinuation of the therapy. Most ADA responses consist of IgG switched antibodies, whose development is facilitated by CD4^+^ helper T (Th) cells. Of note, Th cells also aid the differentiation of effector cytotoxic T lymphocytes (CTL) that i.e., originating from gene therapy interventions, can eliminate target cells transduced with the therapeutic transgene. Th cells are essential regulating the immune response against biotherapeutics. Accordingly, evaluating Th cell mediated immunity is a critical component of immunogenicity assessments (9–11).

Comprehensive evaluation of cellular responses in clinical settings is challenging due to the intricacy of cellular immunity and the variability of individual immune responses (12–14). Enzyme-Linked ImmunoSPOT (ELISPOT), has been the gold standard for assessing drug-induced cellular responses. ELISPOT assays are labor-intensive, have limited sensitivity and classically measure only one (maximum three) analyte(s) at a time, in the absence of any phenotypic information, thus offering limited insights into the complexity of the immune response (15–20). Consequently, there has been a need for developing techniques to efficiently detect and characterize cellular responses to biotherapeutics in a robust and simple manner. Furthermore, because many biotherapeutics do not exhibit cross-reactivity with targets from distant species to human, non-human primates (NHPs) are typically the preferred, and often the only viable species for preclinical testing and safety evaluations. This preference introduces additional challenges to the preclinical determination of immunogenicity responses due to the scarcity of protocols that effectively work with NHP cells.

Activation-Induced Marker (AIM) assays are gaining popularity over ELISPOT for the assessment of antigen-specific T cell recall responses (9, 21). AIM assays not only enable the detection and quantification of antigen-specific T cell activation but permit a simultaneous phenotype of the responding cell population (9, 21–28). Recently, AIM assays have been expanded to measure the co-expression of several pairs of activation markers. This advancement permits a more comprehensive and nuanced analysis of cellular responses (29). While AIM assays are validated for measuring T cell responses in humans, studies utilizing these assays on specimens from NHP are limited, typically concentrating on the detection of CD25 and CD134 expression (30–32). As a result, these assays may not comprehensively capture the full breadth of T cell responses, nor provide extensive information about the phenotypic context in which these responses occur. The limited development of NHP-validated AIM assays may be attributed to the specific challenges associated to the work with this species, particularly the high study costs and the restricted blood volumes available for analysis. The latter constraint is also relevant to clinical pediatric subjects, where minimally invasive and efficient sampling methods are imperative (33). Therefore, optimizing AIM assays to accurately measure T cell responses in NHPs, based on the expression of multiple AIM pairs and using small blood volumes, is important for both preclinical research and translation to clinical settings.

Here we present a flow-cytometry-based composite AIM (cAIM) assay designed to proficiently measure antigen-specific T cell responses in peripheral blood of cynomolgus monkeys. The assay was initially developed in a fluorescent cytometry platform using Keyhole Limpet Hemocyanin (KLH) as the model antigen and allowed the simultaneous detection of up to 10 pairs of AIMs, facilitating the quantification of antigen-specific CD4^+^ and CD8^+^ T cell recall responses in peripheral blood mononuclear cells (PBMCs). Subsequently, we evaluated the capacity of the cAIM assay to identify AAV9-specific T cell responses in PBMCs from human donors who were not exposed to gene therapy products but were sero-positive for multiple AAV variants. Using *in-silico* and *in vitro* tools, we identified a cocktail of 27 peptides derived from the AAV9 capsid proteins suitable to detect human T cell recall responses and used it to evaluate AAV9-specific T cell responses in NHPs as well. The cAIM assay was then evaluated in the mass-cytometry platform, using cytometry of time of flight (CyTOF), which allowed simultaneous broad immunophenotyping and could be performed directly on whole blood.

Our cAIM assay serves as a robust tool for evaluating antigen-specific T cell recall responses to various antigens in cynomolgus monkeys and humans. The assay runs on the fluorescent or mass cytometry platform, measures multiple AIM pairs and uses composite parameters (cAIM-index and cAIM-score) to summarize responses into quantitative metrics, aiding interpretation and comparison across samples and conditions. We anticipate that the protocols described herein will facilitate the preclinical and clinical assessment of immunogenicity to biotherapeutics and hence, advance the development of novel therapeutic modalities.

## Results

### 6x cAIM assay monitors kinetics of antigen-specific CD4^+^ T cell recall response to KLH in NHPs

**Figure 1:**
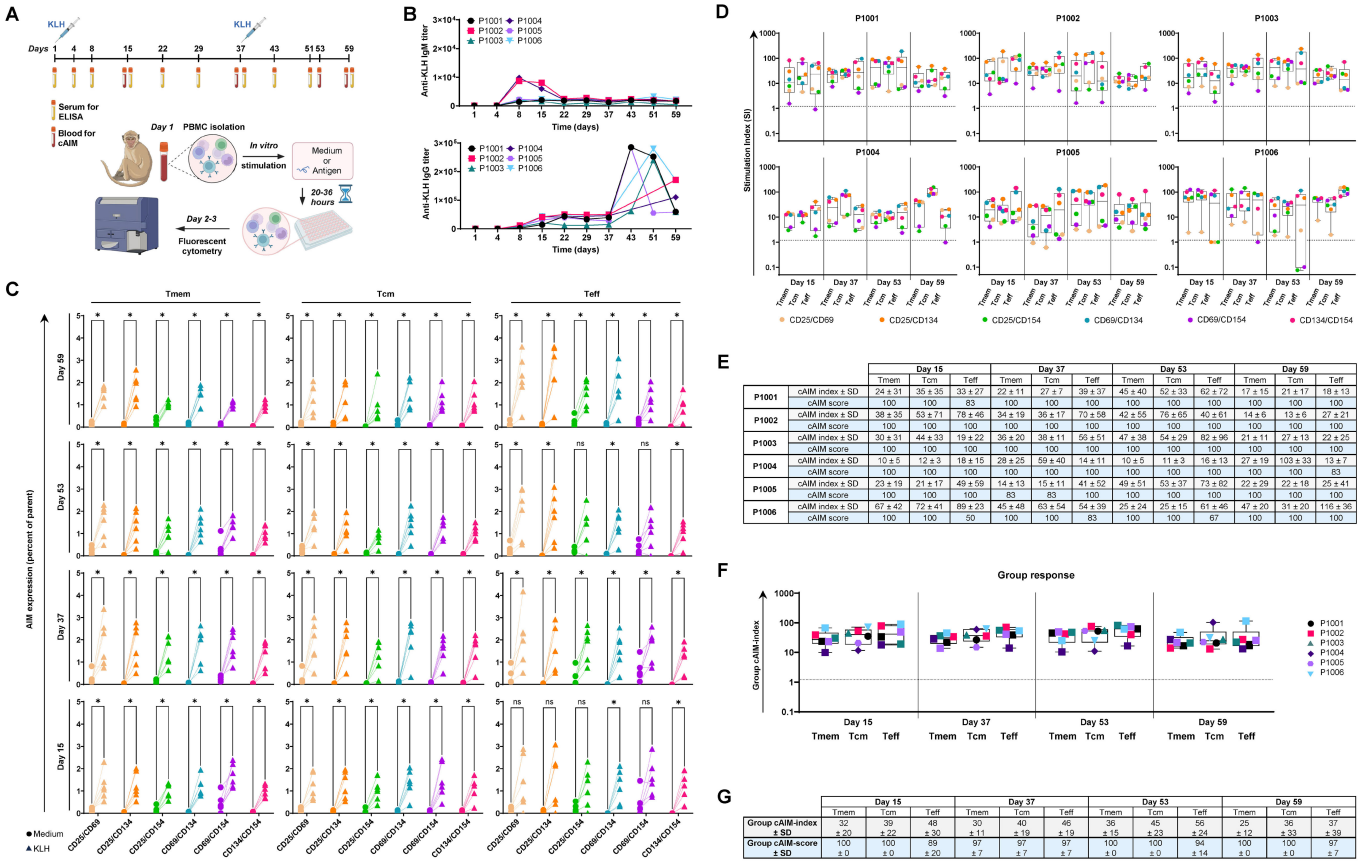
Establishment of a 6x cAIM assay to assess KLH-specific CD4^+^ T cell responses in cynomolgus monkeys: **(A)** Animals were immunized with KLH on days 1 and 37. Blood samples for antibody titration (x10) and AIM assays (4x) were obtained at the indicated time points. Freshly isolated PBMC were cultured in medium alone (control) or containing KLH (stimulated) for up to 36 hours and antigen-specific activation of CD4^+^ T cells was determined by measuring the combinatory surface expression of CD25, CD69, CD134 and CD154 using fluorescent cytometry. Created in BioRender. Schmidt, J. (2025) https://BioRender.com/f8faxbu. **(B)** Kinetics of anti-KLH, IgM and IgG antibody response. **(C)** Expression level of AIM pairs within the CD4^+^ T cell memory (Tmem), central memory (Tcm) and effector memory (Teff) compartments. PBMC, isolated at the indicated days, were cultured in medium alone (circles) or containing KLH (triangles). Paired control-stimulation expressions of marker combinations for each animal are connected by solid lines. Significance of the differences in AIM expression from control versus stimulated samples of all animals and CD4^+^ T cell subsets was evaluated by the Mann-Whitney test using the Holm-Šídák method (alpha = 0.05). P-values >0.05 (ns) and ≤0.05 are denoted as (ns) and (*), respectively. **(D)** Kinetics of AIM responses expressed as SI in all CD4^+^ T cell subpopulations. Boxplots indicate median values and 75^th^ and 25^th^ percentiles. Whiskers extend from the lowest to highest AIM data point. **(E)** cAIM-indices (average of all the SI ≥1.2) and cAIM-scores (percentage of AIM combinations with SI ≥1.2). **(F)** Group response to KLH, based on the average cAIM-indices (group cAIM-index) of the subjects presented in **(D)**. **(G)** Group cAIM-index of and group cAIM-score of the individual cAM response reported in **(D)**, indicating the corresponding standard deviation (SD) value.

To address the challenge of comprehensively and robustly detecting antigen-specific T cell responses in NHPs, we developed a cAIM assay by measuring the simultaneous expression of six pairs of activation markers: namely, the combinatorial expression of CD25, CD69, CD134 (OX40), and CD154 (CD40L) (**Supplementary Table 1**). We established our cAIM assay by characterizing the CD4^+^ T cell response to KLH given its known immunogenic properties, good safety profile, and widespread use in immunological research (34). To this end, we immunized six animals with 10 mg of KLH on days 1 and 37 using a subcutaneous (SC) route of immunization and conducted regular blood sampling throughout the study (**Figure 1A**).

Immunization with KLH induced an anti-KLH IgM response in all exposed subjects (**Figure 1B**), peaking on day 8 and displaying titer values up to 10,000. Titers decreased slightly until day 22 and then stabilized around 2,000-2,500. Subject P1003 exhibited the lowest IgM response, with a peak titer of 1,850 on day 8, which dropped to 450 by day 59. Administering a second dose of KLH on day 37 did not induce a further increase in anti-KLH IgM titers. In contrast to anti-KLH IgM, the anti-KLH IgG antibody response exhibited a dual peak pattern (**Figure 1B**). The initial peak, with titers reaching up to 50,000, occurred between days 15 and 22. After this period, the anti-KLH values remained relatively stable until the second immunization on day 37, which led to further increase in titers, up to 285,000, between days 43 and 51. These values decreased two- to five-fold a week later in most animals. Overall, we detected robust anti-KLH IgM and IgG humoral responses in all study animals, consistent with the well characterized immunogenic nature of KLH. The elevated titers of IgG switched anti-KLH antibodies after the second immunization with KLH, were indicative for a strong Th cell support.

In alignment with the observed humoral response, the cAIM assay revealed a robust antigen-specific increase in the expression of all assessed AIM pairs (CD25/CD69, CD25/CD134, CD25/CD154, CD69/CD134, CD69/CD154 and CD134/CD154) within the total memory (Tmem), central memory (Tcm), and effector (Teff) compartments of CD4^+^ T cells following *in vitro* stimulation with KLH (**Figure 1C**). The overall increase in AIM positive cells was significant for all six AIM combinations in the Tcm compartment from day 15 onwards. The response was also notable among Teff cells although some marker combinations did not achieve significance in some animals on days 15 and 53 (**Figure 1C**). Overall, the Tmem cell response remained significant for all AIM combinations through all experimental days, with percentages of AIM positive cells often exceeding 2% of the parental cell gate, suggesting a durable Th cell response.

To accurately evaluate the overall T cell recall response, we normalized the observed percentages of all activation pairs by determining the fold-change in the stimulated condition compared to the corresponding unstimulated control, commonly referred to as the stimulation index (SI). Because SI cannot be calculated when the percentage of positive events in the control response is zero, we first selected the minimal observed expression value greater than zero for each activation marker pair and added it to all observations within such activation marker combination before calculating the individual SI. This normalization ensured comparability across AIM pairs and prevented division-by-zero artifacts. The SI of all activation markers for the different cell populations and animals was subsequently summarized using boxplots representing the cAIM responses for the different samples (**Figure 1D**). To better compare responses across multiple samples, we defined two composite metrics: the cAIM-index and the cAIM-score. (**Figure 1E**). The cAIM-index was obtained by averaging the SI values of the different AIM pairs, for each T cell subpopulation and date of individual animals, that meet or exceed a positive threshold of 1.2. This threshold was determined a priory, based on extensive laboratory experience ensuring high sensitivity for detecting true antigen-specific responses while maintaining a false-positive rate of less than 0.0001% (see Materials and Methods section). The cAIM-score was defined as the percentage of AIM pairs within each test that fulfilled the SI ≥1.2 criterion (**Figure 1E**). Thus, the cAIM-index reflects the magnitude of antigen-induced T cell activation, while the cAIM-score provides a measure of the consistency and breadth of responses across different AIM pairs. This dual approach increases robustness against variability in individual markers and reduces the risk of underestimating biologically meaningful but modest responses. In contrast to classical AIM assays that focus on a limited marker set, the cAIM framework integrates multiple independent readouts into composite measures, thereby improving reproducibility and confidence in identifying antigen-specific T cell recall responses.

The overall cAIM analysis on the animals immunized with KLH indicated the development of a strong CD4^+^ T cell response following the first immunization (**Figure 1F**) The group cAIM signals remained relatively stable from day 15 and increased slightly after the second immunization, reaching a median group cAIM-index of 36 ± 15, 45 ± 23 and 56 ± 24 in the Tmem, Tcm and Teff cell populations by day 53. Thereafter, the group cAIM-index initiated a decline in all T cell populations, possibly reflecting the contraction of the immune response. The group cAIM-scores were very high across cell subpopulations and kinetics (**Figure 1F**) indicative of a consistent recall response.

Taken together, our 6x cAIM assay and associated cAIM metrics enabled us to confidently monitor the kinetics of the KLH-specific CD4^+^ T cell recall response in the individual and group NHP population, highlighting the contribution of the different Tcm and Teff cell populations.

### cAIM detects AAV9-specific T cell responses in mice and humans

Following the implementation of the 6x cAIM assay for quantifying KLH-specific CD4^+^ T cell responses, we aimed to broaden its applicability by measuring CD4^+^ and CD8^+^ T cell responses to viral antigens, particularly to components of the capsid of the Adeno Associated Virus 9 (AAV9), which is a common vector used for gene therapy. We performed a preliminary experiment in mice, immunizing three C57B6/J mice intravenously (IV) with an AAV9 vector carrying control transgene reporters (**Supplementary Figure 1A**). Cell suspensions from splenocytes were stimulated with medium control or empty AAV9 capsids and *in vitro* antigen-specific CD4^+^ and CD8^+^ T cell recall responses were measured based on the expression of ten different AIM pairs (anti-CD137 [4-1BB] and anti-CD278 [ICOS] antibodies, were included in the cAIM assay). Weak but consistent CD4^+^ and CD8^+^ Tmem, Tcm, and Teff cell responses to the AAV9 capsids were detected, in all tested animals (**Supplementary Figure 1B**). CD4^+^ T cell responses were very homogenous but displayed low SI. CD8^+^ T cell responses were stronger, particularly in the effector compartment and in AIM combinations of the newly introduced CD137 and CD278 markers (**Supplementary Figure 1B**). Overall, these data indicated that our 10x cAIM assay and composite metrics enable the quantification of both helper and cytotoxic T cell recall responses specific to AAV9 in mice (**Supplementary Figure 1C**), and that the inclusion of CD137 and CD278 markers adds additional value to the assay.

The mouse experiment also revealed the challenges of detecting AAV9-specific T cell responses using empty capsids. These structures have the advantage of containing the majority of potential T cell epitopes involved in an immune response to a therapeutic AAV capsid. However, the capsids are large and require time for efficient processing by Antigen Presenting Cells (APCs) prior presentation of the relevant epitopes to T cells, which may compromise the sensitivity of the response in short term assays. Therefore, we decided to evaluate AAV9-specific T cell responses using immunogenic peptides derived from the AAV9 Viral Protein 1 (AAV9-VP1). AAV9-VP1 (UniProt Accession #Q6JC40) is a 736 amino acid protein that contains the splicing variants VP1, VP2 and VP3, which together, in a ratio of 1:1:10, form the capsid of AAV9. The T cell epitopes of AAV9-VP1 are not well defined and classical approaches to identify them require the use of pools of peptides (e.g., 15-mers overlapping by 12 amino acids, spanning the entire VP1 sequence) to test specific T cell activation using PBMCs from individuals seropositive for AAV9. Unfortunately, access to blood from patients treated with gene therapy products is very limited and the frequencies of AAV9 capsid-specific T cells circulating in healthy individuals, which could have been naturally exposed to AAV9 virus is unknown. These challenges prompted us to perform an *in-silico* mapping of AAV9-VP1, searching for peptide sequences predicted to have a high probability of binding to the most common HLA-DR receptors. This strategy could select a limited number of peptides and consequently reduce the number of tests, facilitating T cell epitope mapping using small volumes of blood. Using the approach described in the Materials and Methods section, we identified 27 peptides, ranging from 14 to 22 amino acids length, predicted to bind to most common human HLA-DR displayed by Caucasian populations (**Supplementary Table 2**). Most of the selected peptides aligned with conserved regions across the amino acid sequences of VP1 from AAV-serotypes 1, 6, 8 and 9, (**Supplementary Figure 2**) suggesting that they might also be useful for measuring T cell recall responses to all these serotypes.

**Figure 2:**
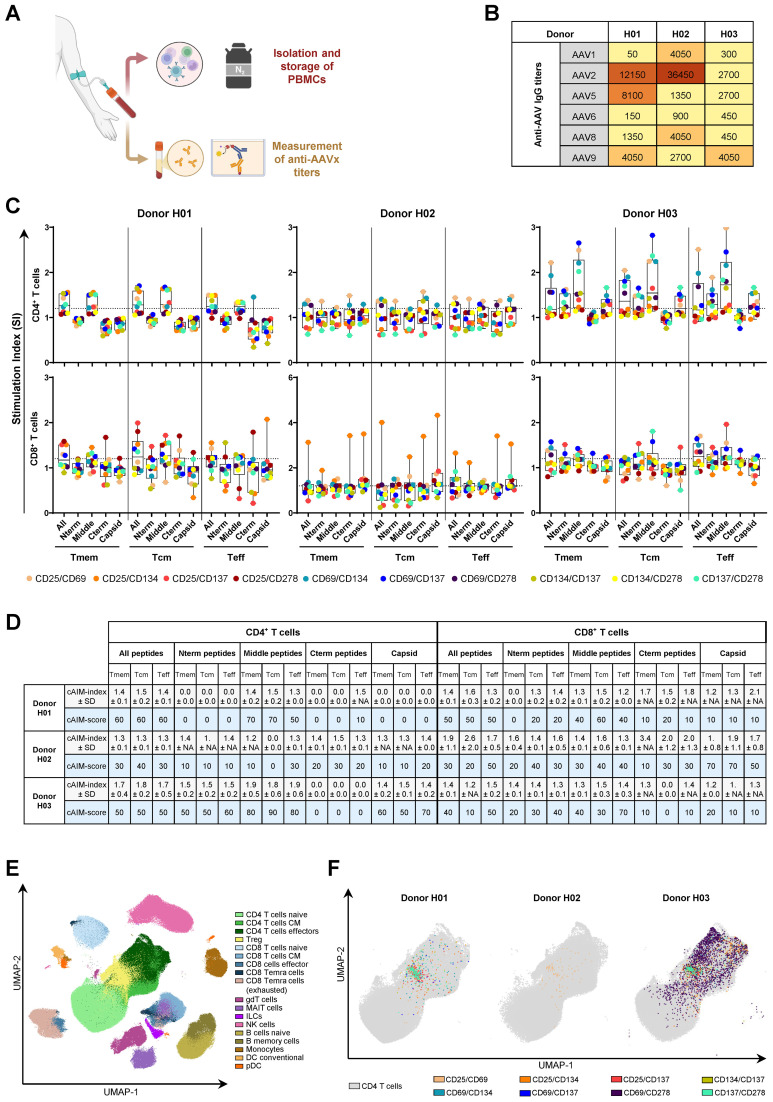
10x cAIM assay enables the characterization of AAV9-specific T cell responses in human donors sero-positive for multiple AAV variants. **(A)** Blood samples from three donors were processed into PBMC and serum. PBMCs were stored frozen for future AIM analysis while serum samples were used to determine AAVx-specific IgG reactivity by ELISA. Created in BioRender. Schmidt, J. (2025) https://BioRender.com/vouwb3y. **(B)** Serum titers of anti-AAV1, 2, 5, 6, 8 and 9. **(C)** AAV9-specific CD4^+^ and CD8^+^ T cell recall responses assessed using the 10x cAIM assay in the mass cytometry platform. Dots represent the individual SI for each AIM marker pair obtained by comparing the stimulation of medium alone (control) to the stimulations with the indicated AAV9 VP1-derived peptide pools or empty AAV9 capsids (**Supplementary Table 2**). Boxplots indicate median values and 75^th^ and 25^th^ percentiles. Whiskers extend from the lowest to highest AIM data point. **(D)** cAIM-indices (average of all the SI ≥1.2) ± standard deviation (SD) and cAIM-scores (percentage of AIM combinations with SI ≥1.2). **(E)** UMAP (51) analysis representing the cell populations detected by mass cytometry on the concatenated samples of the three donors. Eighteen unique cells clusters were identified in the dimensionally reduced data using PhenoGraph (52) by utilizing the median expression values for each marker as a basis to assign the different cell types. **(F)** Mapping of CD4^+^ T cells contributing to the individual AIM indices (SI ≥1.2) following stimulation with the middle (322-456) pool of AAV9 VP1-derived peptides.

Thereafter, we selected frozen human PBMCs from three donors who, although naïve to AAV-based gene therapy, displayed IgG sero-reactivity to AAV1, AAV2, AAV5, AAV6, AAV8 and AAV9 (**Figures 2A, B**). We conducted a cAIM assay on those PBMCs using whole empty AAV9 capsids, the cocktail of the 27 AAV9-VP1 peptides previously selected, and additional sub-pools of the 27-peptide cocktail spanning immunogenic areas in the N-terminus (7-270), middle region (322-456) and C-terminus (456-722) of AAV9-VP1 (**Supplementary Table 2**). Furthermore, we tested the feasibility of moving the readout platform from fluorescent cytometry to mass cytometry using a CyTOF XT instrument.

Following stimulation with the indicated antigens, we evaluated the expression of ten different AIM pairs on CD4^+^ and CD8^+^ T cells by staining the PBMCs with the Maxpar^®^ Direct™ Immune Profiling Assay™ combined with the Maxpar^®^ Direct™ T Cell Expansion Panel 3 (**Supplementary Table 3**). We used these predefined mass cytometry panels due to their high degree of validation and user-friendliness. Consistent with the observed inter-donor variations in the serum anti-AAV IgG titers, our AIM assay displayed a donor variable T cell recall response to the AAV9-derived antigens (**Figure 2C**). The AAV9-specific CD4^+^ T cell response in Donor H02 was poor, yielding most SI below 1.2, as noted by the consistent low cAIM-scores (**Figure 2D**). Notably, this donor exhibited very high antibody titers against AAV2 capsids but displayed the lowest response for the AAV9 serotype (**Figure 2B**). In contrast, Donor H03, who had low antibody titers against all AAV variants except AAV9, displayed strong CD4^+^ T cell recall responses upon stimulation with all AAV9-derived antigens, apart from the C-terminal peptide pool. On the other hand, Donor H01, who had relatively high sero-reactivity for AAV2 and AAV5 and moderate for AAV9, also displayed moderate T cell recall responses to the AAV9 antigens, particularly to the peptide pool spanning amino acids between positions 322 and 456 (**Figures 2C, D**). The CD8^+^ T cell responses were more heterogenous and of lower intensity than those observed for CD4^+^ T cells, probably because the peptides were selected on Class II MHC and due to their length require cross-presentation for activation of CD8^+^ T cells. Nevertheless, Donors H01 and H03 displayed some reactivity with highest SI in response to the pool of peptides covering amino acids 322-456 (**Figures 2C, D**). Overall, CD8^+^ T cells did not respond to empty capsids or did so very weekly and driven mostly by the expression of one single AIM combination (CD25/CD134). Taken together, our data suggest that the selected peptide pool, particularly the peptides located in the middle region and N-terminal regions of AAV9-VP1 contain immunogenic T cell epitopes that drive AAV9-specific CD4^+^ and, to a certain extent, CD8^+^ T cell responses.

Running the cAIM on the CyTOF platform allows for an expanded phenotypic analysis beyond the classical subsets of naïve, central memory, and effector memory CD4^+^ or CD8^+^ T cells by classifying them according to other markers like their chemokine receptor expression. Additionally, this approach enables a simultaneous examination of other leukocyte populations, including gamma/delta T cells, ILCs, MAIT, NK cells, B cells, monocytes, dendritic cells, and plasmacytoid dendritic cells. The relative distribution of these populations can be illustrated using dimensional reduction techniques such as Uniform Manifold Approximation and Projection (UMAP) (**Figure 2E**). Once the UMAP is generated, AIM expression can be overlaid onto the CD4^+^ T cells (**Figure 2F**). This visualization highlights the preferential association of specific AIM markers with distinct CD4^+^ T cell subsets. By resolving these subsets within the UMAP, the analysis gains granularity, revealing how activation is spatially and phenotypically localized across functionally distinct populations.

### 10x cAIM fluorescent cytometry assay identifies CD4^+^ T cell recall responses against KLH, OVA, and AAV9 vectors in NHP

Building on the successful detection of AAV9-specific T cell responses in humans, we aimed to measure T cell recall responses to AAV9 in NHPs. Additionally, we sought to evaluate the effectiveness of the cAIM assay in assessing CD4^+^ T cell reactivity to an antigen with low immunogenicity, specifically chicken Ovalbumin (OVA). We decided to include KLH as positive control in this study and tested OVA alone or together with the alum-based adjuvant Alhydrogel to ensure robust OVA-specific T cell responses. We utilized a slightly different panel than in the studies with KLH alone, i.e., on top of CD25, CD69, CD134 and CD154 we added CD137, in replacement of CD127 (**Supplementary Table 4**).

**Figure 3:**
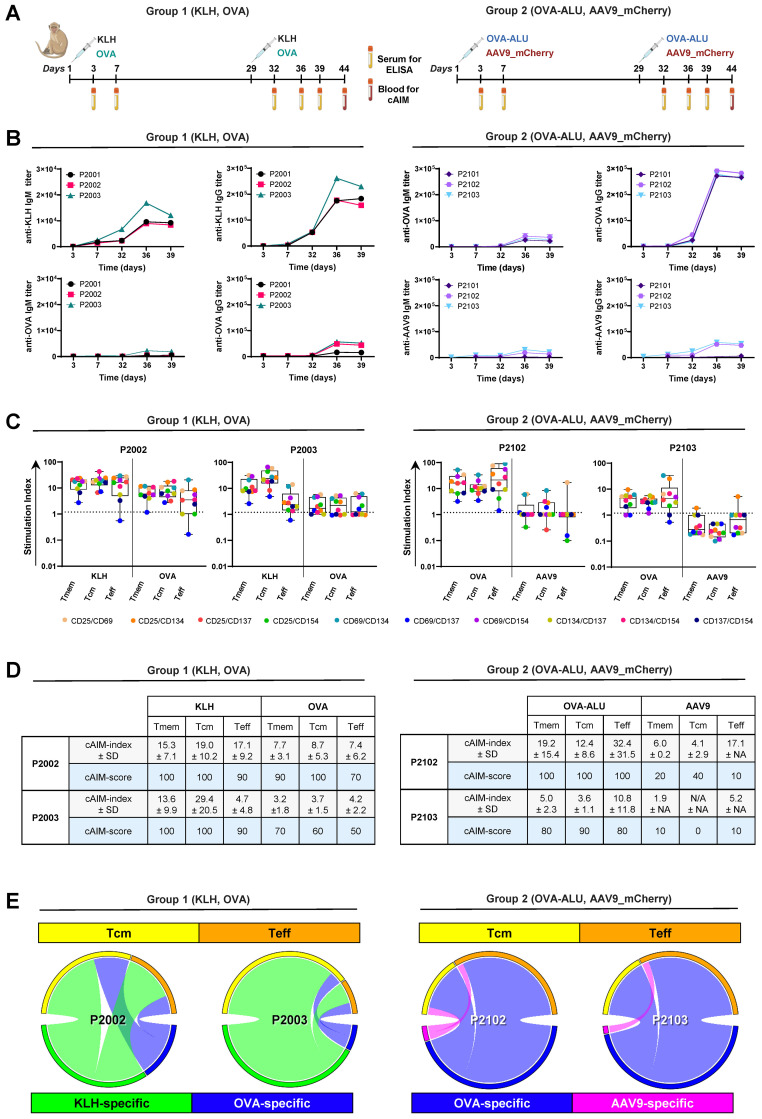
Detection of KLH-, OVA- and AAV9-specific CD4^+^ T cell responses in cynomolgus monkeys using a 10x cAIM assay. **(A)** Design of *in vivo* study. Six cynomolgus monkeys were split into two groups and dosed with either KLH and chicken OVA (Group 1) or with OVA in Alhydrogel and AAV9-mCherry vectors (Group 2) on days 1 and 32. Blood samples for antibody titer determination were obtained from all animals throughout the study. Blood samples for PBMC isolation and AIM assays were obtained from animals P2002–3 and P2102–3 on day 44. Created in BioRender. Schmidt, J. (2025) https://BioRender.com/29digcn. **(B)** Kinetics of anti-KLH, anti-OVA and anti-AAV9 IgM and IgG production in all animals. **(C)** Kinetics of AIM responses expressed as SI in all CD4^+^ T cell sub-populations of animals P2002–3 and P2102-3. Boxplots indicate median values and 75^th^ and 25^th^ percentiles. Whiskers extend from the lowest to highest AIM data point. **(D)** cAIM-indices (average of all the SI ≥1.2) ± standard deviation (SD) and cAIM-scores (percentage of AIM combinations with SI ≥1.2). **(E)** Circular plots illustrate the relative contribution of the central memory and effector T cell population to the total AIM response to the different antigens (Group1: KLH [green], OVA [blue]; Group2: OVA [blue], AAV9 [pink]) in the individual animals. Arcs are constructed as a product of cAIM-indices and corresponding cAIM-scores.

Six NHP were randomized into two groups of three animals each. Group 1 received a SC dose of 10 mg KLH and 5 mg OVA on days 1 and 32. Group 2 received a SC injection of 5 mg OVA in Alhydrogel (OVA: Alu, 1:1) and an IV injection of AAV9 vectors (2x10^12^ vg/kg) encoding a control mCherry transgene on days 1 and 32. We collected serum samples from all animals throughout the study and sampled blood from two animals of each group (P2002, P2003 and P2102, P2103) for cAIM analysis on day 44 (**Figure 3A**).

After the second immunization, all animals exhibited detectable IgM and IgG antibody responses to the different antigens (**Figure 3B**). However, the titer levels and the heterogeneity of the responses varied depending on the specific immunization protocol. IgM and IgG antibody titers were particularly elevated (maximum IgG titers >200,000) in animals challenged with KLH or OVA/Alu. The antibody responses elicited by KLH or OVA: Alu were also homogenous across the different animals whereas humoral responses to OVA alone or AAV9 vectors displayed high group variability. OVA-specific titers in animals receiving OVA alone were moderate (maximum IgG titers around 50,000 only in two of three animals; P2002 and P2003). AAV9 specific titers in animals exposed to the AAV9 vectors were also moderate (maximum IgG titers around 50,000 and only in two of three animals; P2102 and P2103). IgG responses to OVA alone or AAV9 vectors in animals P2001 and P2101, respectively were low displaying OVA-specific IgG titers of 16,000 and AAV9-specific IgG titers of 6000 (**Figure 3B**).

Consistent with the observed antigen-specific antibody titers, the extent of *in vitro* CD4^+^ T cell recall responses to KLH or OVA were noticeably upregulated in PBMCs from animals immunized with either KLH or OVA mixed with Alhydrogel (**Figures 3C, D**). OVA-specific CD4^+^ T cell responses were also detected in stimulated cultures of PBMCs isolated from animals exposed to OVA alone, although the responses were lower compared to those observed in animals immunized with OVA and Alhydrogel. The AAV9-specific T cell recall responses, observed in animals from Group 2 after *in vitro* stimulation with the AAV9-peptides, selected according to their predicted HLA-binding, did not significantly exceed background levels. Only exceptional moderate increases in the percentage of cells expressing CD25/CD69, CD69/CD134 (P2102) or CD25/CD134 (P2103) were detected (**Figure 3C**, **Supplementary Figure 3**). The restricted CD4^+^ T cell response to these three AIM pairs as well as the overall low-level reactivity following *in vitro* stimulation with AAV9 was unexpected but probably attributed to the low dose of AAV9 vectors selected for immunization. Although the immunization with 2 x 10^12^ vg/kg AAV9 vectors was sufficient to elicit moderate IgG antibody titers, the dose was ten-fold lower than that used in the mouse experiment (2.5 x 10^13^ vg/kg) and 40 to 100-fold below the doses commonly used in gene therapy protocols for human (35).

In addition to the differences triggered by the individual antigens, our composite 10x cAIM assay revealed variations of reactivity among the different T cell compartments (**Figures 3C–E**). KLH responses tended to concentrate in the central memory compartment, whereas OVA specific responses were equally distributed between central and effector memory subsets in animals immunized with OVA alone or concentrating in the effector arm in animals receiving OVA together with adjuvant. The few responses detected following stimulation with AAV9 peptides were slightly skewed towards the Teff cell compartment. (**Figure 3E**). In general, predominant compartmentalization of an immune response in central memory T cell subsets are indicative of long-term immune surveillance and the potential for sustained recall capacity, whereas effector-dominated T cell responses are more characteristic of active immune engagement (36–38).

Overall, our findings underscore the capability of the composite 10x cAIM fluorescent cytometry assay in distinguishing variations in CD4^+^ T cell recall responses across different T cell subsets and antigens with varying levels of immunogenicity in NHPs.

### Mass cytometry cAIM assay detects antigen-specific T cell responses in whole blood of NHPs

**Figure 4:**
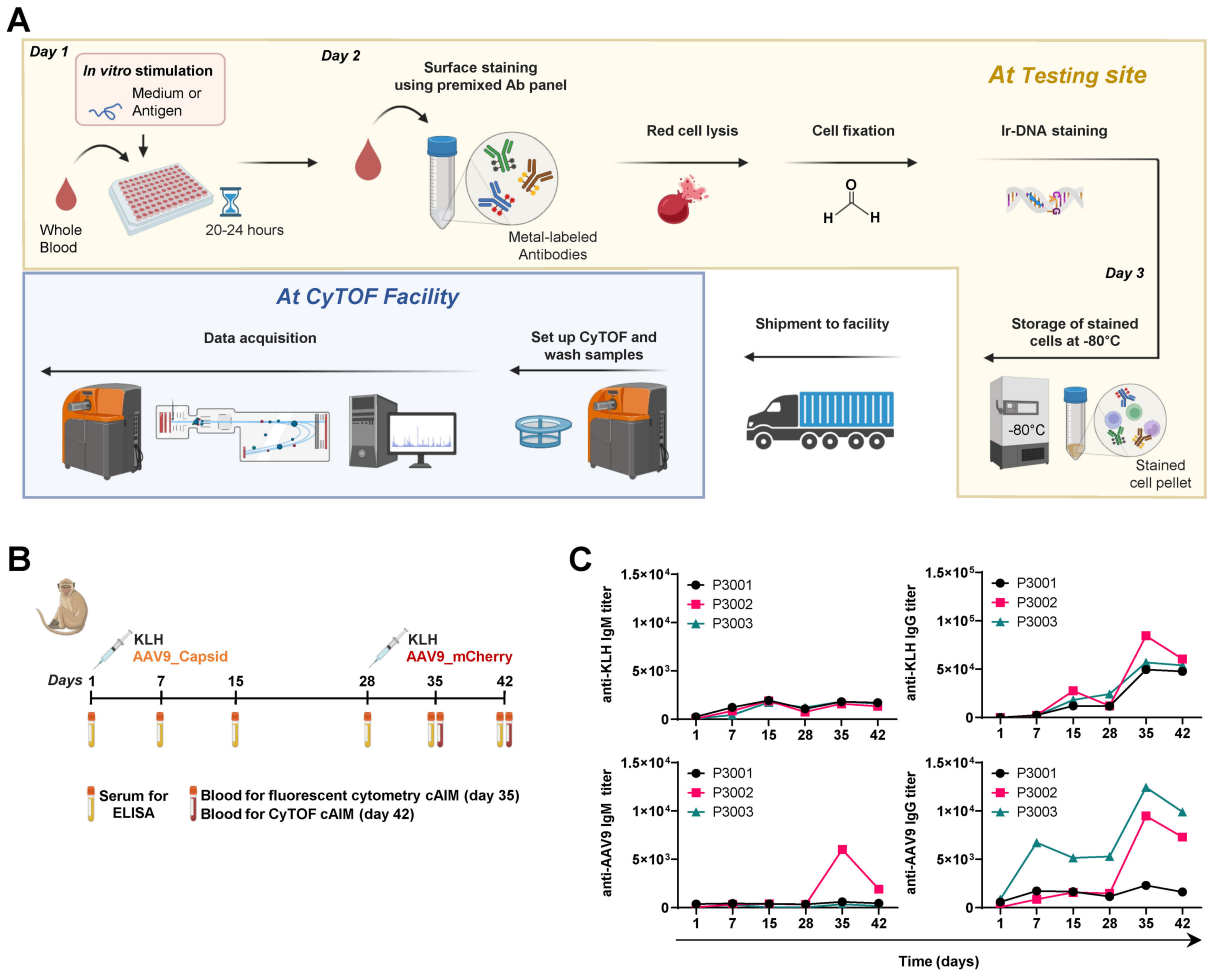
Overview of the cAIM mass cytometry assay. **(A)** After collection, whole blood samples are transferred directly into cell culture plates and left unstimulated or stimulated with the antigen of choice for 20–24 hours. Subsequently, blood cells are directly stained with a cocktail of antibodies labelled with monoisotopic metals. Erythrocytes are lysed and samples are fixed and stained with iridium overnight. Stained cell pellets can be then either processed without delay or stored at -80°C for up to several months, with the possibility to ship them to a different location (CyTOF facility). Created in BioRender. Schmidt, J. (2025) https://BioRender.com/oom9qt5. **(B)** Design of *in vivo* study. Three cynomolgus monkeys were immunized with KLH and empty AAV9 capsids on day 1 and with KLH and AAV9-mCherry vectors on day 28. Blood samples for antibody determination were obtained from all animals throughout the study. Blood samples for AIM assays were obtained on day 35 (and processed into PBMCs for fluorescent assays) and on day 42 (without cell separation for mass cytometry assays). Created in BioRender. Schmidt, J. (2025) https://BioRender.com/yiu61oz. **(C)** Kinetics of anti-KLH and anti-AAV9 IgM and IgG titer production in all study animals.

Preclinical research involving NHPs is frequently outsourced to contract research organizations (CROs) due to the needs for specialized facilities and regulatory compliance. These studies are inherently complex and require the involvement of various teams to treat and conduct *in vivo* evaluations of the animals and collect numerous samples throughout the duration of the study for processing and analysis in specialized *in vitro* laboratories. The maximum allowable blood volumes that can be drawn from these animals are regulated strictly. The limitations on blood volumes, combined with the procedures for isolating, freezing, and thawing PBMCs prior to *in vitro* analysis, may influence the ability to detect antigen-specific T cell responses. To address these challenges, we sought for a method to robustly assess antigen-specific T cell recall responses using small blood volumes. The process should start immediately after sample collection, avoid lengthy manipulations and provide the flexibility to pause it at certain points and resume it when laboratory routines are less busy. These considerations led us to the implementation of a cAIM assay directly on whole blood using the mass cytometry platform (**Figure 4A**). Typically, only 1.5 mL of blood are required to perform a cAIM assay in response to three different challenges (i.e., negative control, positive control and test antigen) in conjunction with a broad immunophenotyping analysis of blood cells. The *in vitro* stimulation is conducted directly on aliquots of whole blood cells, having the possibility of preparing tubes preloaded with stimulatory antigens. The process can be stopped after *in vitro* stimulation and sample staining with monoclonal antibodies labelled with monoisotopic metal tags (**Supplementary Table 5**). The stained pellets can be frozen and stored for months before acquisition in a CyTOF instrument at the same or at a remote facility (**Figure 4A**). This approach is particularly advantageous for studies involving a large number of samples or longitudinal studies, as it enhances workflow flexibility, allows acquisition on kinetic samples on the same day, and facilitates the efficient allocation of resources (39, 40).

Building on the advantages of the mass cytometry platform, we collected blood samples from animals that had received two SC injections of KLH (10 mg/mL per dose on days 1 and 28) and one IV injection of empty AAV9 capsids (1x10^13^ vp/kg on day 1), followed by a booster IV injection with AAV9-mCherry vector (1x10^13^ vg/kg on day 28) (**Figure 4B**). The unprocessed whole blood was divided into three aliquots and each of them exposed for a 24-hour stimulation to medium containing 0.25% DMSO (negative control), KLH (positive control) or the pool of peptides derived from AAV9-VP1 (test antigen). As described in the Material and Methods section, the samples were frozen shortly after staining and subsequently shipped overseas for acquisition in a CyTOF instrument at a distant facility (**Figure 4A**).

Overall, the immunization with the two different antigens elicited measurable IgM and IgG antibody responses (**Figure 4C**). However, the magnitude of the responses was substantially lower in comparison to previous studies (**Figures 1B**, **3B**). Determining the reasons for this reduced response is challenging and may be attributed to the inherent variability of immune responses commonly observed when small groups of NHP (especially coming from different colonies) are studied. Anti-KLH IgM production showed a double wave pattern, with the first peak on day 15 (titers around 2,000) followed by a second peak on day 35 with a slightly lower signal intensity. Anti-KLH IgG titers topped after the second immunization on day 35, with animal P3002 exceeding values of 80,000 and about 60,000 for the other two animals. The antibody titers against AAV9 displayed a high variability. Only animal P3002 displayed a weak but noticeable IgM response and only after the second immunization (titer value 6,000). Anti-AAV9 IgG titers were detected for animals P3002 and P3003 with maximal peaks of 9,000 and 12,500 values, respectively on day 35. Humoral IgM and IgG responses for animal P3001 were minimal and displayed a maximal titer for anti-AAV9 IgG of 2,300 on day 35.

Half of the antibodies used in the CyTOF analysis were not readily available at the vendor site and had to be labelled in-house. This prompted us to perform a simultaneous cAIM test using the fluorescent cytometry method described earlier in comparison to the planned mass cytometry experiments. The availability of metal-labeled antibodies also limited us to six antibody combinations for the mass cytometry cAIM, although on the other hand we could include a large panel of phenotyping markers. Additionally, technical constraints prevented collecting sufficient blood to perform both fluorescent and mass cytometry experiments on the same day. As a result, the fluorescent cytometry cAIM test was done with PBMCs isolated from blood drawn on day 35 while the mass cytometry cAIM assay was conducted with fresh blood collected on day 42 (**Figure 4B**).

**Figure 5:**
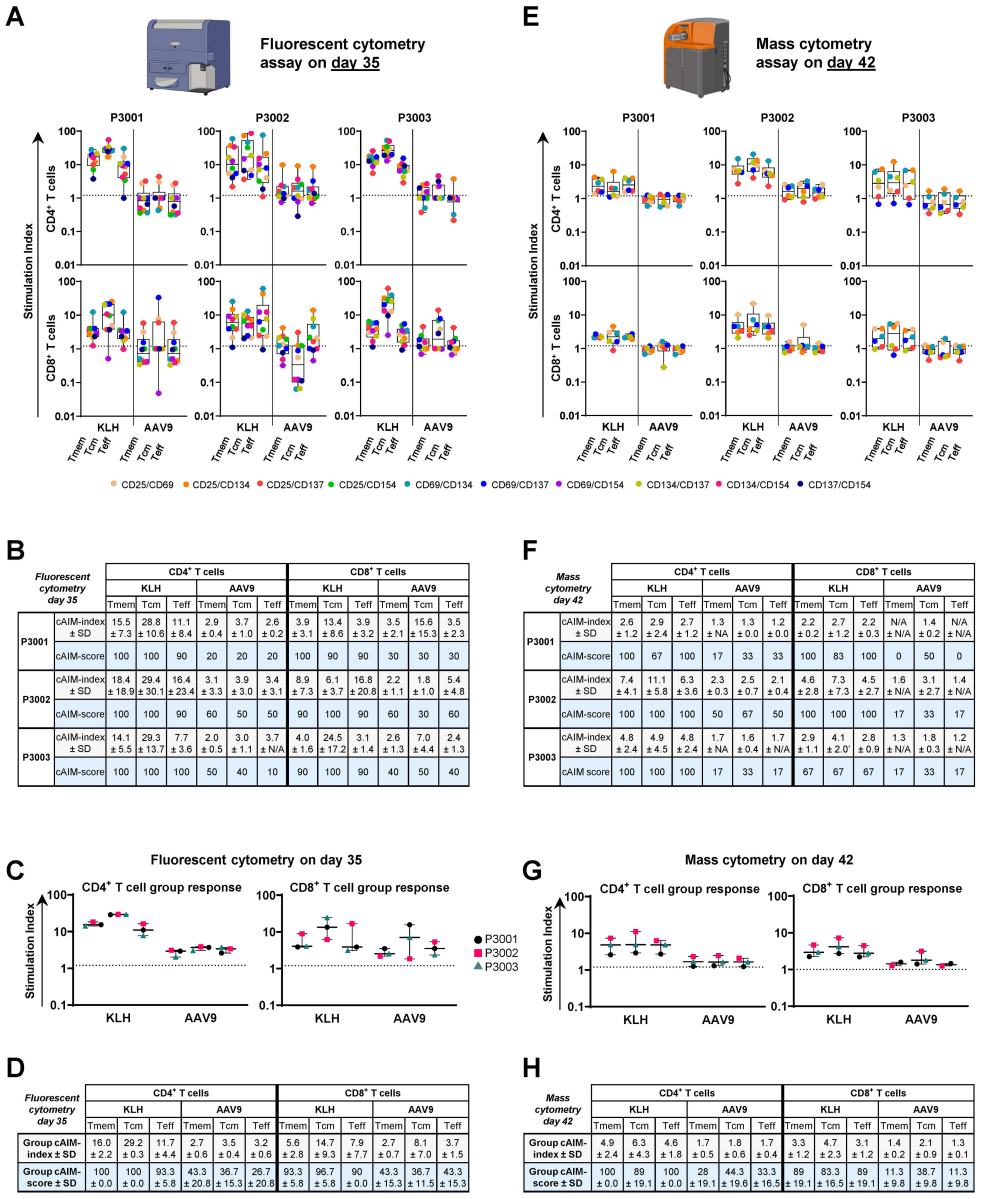
Mass cytometry cAIM assay to measure antigen-specific T cell responses in whole-blood samples from cynomolgus monkeys. **(A, E)** Kinetics of cAIM responses using the fluorescent **(A)** or mass cytometry **(E)** platform. Data is expressed as SI in all CD4^+^ and CD8^+^ T cell subpopulations of the study animals. Boxplots indicate median values and 75^th^ and 25^th^ percentiles. Whiskers extend from the lowest to highest AIM data point. Created in BioRender. Schmidt, J. (2025) https://BioRender.com/u6liqrc and https://BioRender.com/w8yrzo5. **(B, F)** cAIM-indices (average of all the SI ≥1.2) and cAIM-scores (percentage of AIM combinations with SI ≥1.2) of data presented in **(A)** and **(E)**, respectively. **(C, G)** Summaries of the overall population response to KLH and AAV9 peptide pool. The boxplots are generated using the average cAIM-indices reported in **(B, F). (D, H)** Group cAIM-index and group cAIM-score with corresponding standard deviation of the AIM response of all subjects reported using the fluorescent **(A)** and mass cytometry **(E)** platforms.

Using the fluorescent-cytometry platform, we detected KLH- and AAV9-specific CD4^+^ and CD8^+^ T cell responses in PBMCs from most animals (**Figures 5A, B**). KLH-specific CD4^+^ T cell recall responses were induced in all three animals upon *in vitro* stimulation. cAIM responses tended to be higher in the central memory compartment compared to the effector populations reaching a group cAIM-index of 29.2 ± 0.3 and group cAIM-score of 100 (**Figures 5C, D**). KLH-specific CD8^+^ T cell responses followed a similar trend but were slightly lower in magnitude. Both CD4^+^ and CD8^+^ T cell responses to AAV9 were lower than those observed in response to KLH, with several AIM pairs showing SI below 1.2 (**Figures 5C, D**). Overall, CD4^+^ and CD8^+^ T cell recall responses to AAV9 were detectable, reaching maximal group cAIM-indices of 3.5 ± 0.4 and 8.1 ± 7 for CD4^+^ and CD8^+^ T cells, respectively, but having low group cAIM-scores (around 40) compared to the robust group cAIM-scores (>90) elicited by KLH antigen (**Figures 5C, D**).

KLH and AVV9-specific T cell responses were also assessed in fresh whole blood samples on day 42 using our newly developed mass cytometry cAIM assay. As observed with fluorescent cytometry evaluation high KLH-specific responses were detected in both CD4^+^ and CD8^+^ T cell compartments (**Figures 5E, F**), with also higher responses by the CD4^+^ T cells. CD4^+^ and CD8^+^ T cell recall responses specific to AAV9 were minimal and only slightly above our defined baseline levels for some marker combinations, suggesting that the cellular response against AAV9 might had already contracted by day 42 (**Figures 5E, F**). Although mass cytometry results are not directly comparable to the fluorescent readouts due to the different time of sample collection, use of whole blood instead of PBMCs, different number of AIM pairs analyzed and, in some cases, different antibody clone for same surface receptors, the outcomes from the two assays were very consistent (**Figures 5C, D, G, H**).

**Figure 6:**
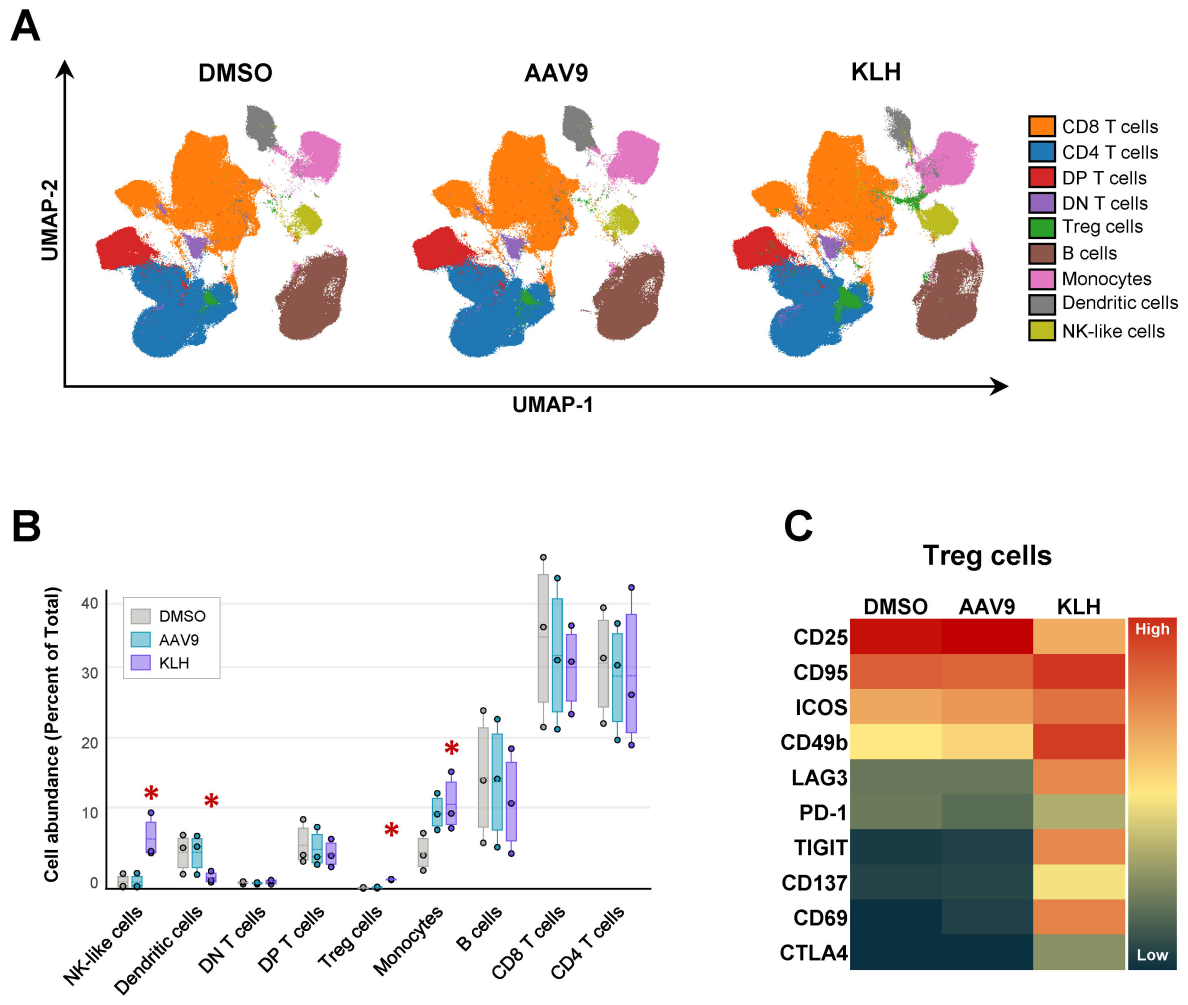
Broad immunophenotype of mononuclear cell populations in cynomolgus whole blood. **(A)** UMAP (51) and FlowSOM (55) analysis of mass cytometry data, using whole blood from the three cynomolgus monkeys reported in **Figure 4** identified 20 unique phenotypes, which were subsequently consolidated into 9 clusters representing different immune cells. **(B)** Influence of control, AAV9 peptide pool- and KLH-stimulation on cluster abundance. Asterix indicates significant changes (p-value ≤ 0.5) calculated using edgeR (56). **(C)** Heatmap of median expression values of surface markers associated to Treg cell differentiation or function in response to T cell activation.

The larger number of analytes used in the mass cytometry panel allowed us to assess additional parameters beyond AIM responses. Using dimension reduction analysis, 20 cell clusters were identified. Based on the combined expression of surface receptors, these clusters were further condensed into 9 clusters likely representing T cells, B cells, NK cells, monocytes, and dendritic cells (**Figure 6A**). When we compared the cellular distribution of concatenated blood samples from all three animals following *in vitro* stimulation, we observed small changes (**Figure 6B**). KLH stimulation led to an increased frequency of Treg and NK cells, along with a rise in monocyte proportions at the expense of the dendritic cell population. Conversely, stimulation with AAV9-VP1 derived peptides led to an increase in monocyte frequency, although this increase was not statistically significant. Notably, the KLH-induced expansion of the Treg cell cluster was accompanied by immunophenotypic changes, as several surface receptors associated with Treg cell development and/or function showed increased signal intensity compared to the control condition (**Figure 6C**).

In conclusion, our mass cytometry cAIM assay effectively detected antigen-specific T cell responses in whole blood from cynomolgus monkeys, addressing prior challenges and bottlenecks connected to sample processing, quality and storage. Moreover, the assay provided substantial insights into antigen-specific cellular responses beyond conventional CD4^+^ T cells.

## Discussion

Monitoring antigen-specific T cell recall responses is critical for evaluating immunological memory and vaccine efficacy. Traditionally, these responses have been assessed using ELISPOT assays, which are limited to detecting one or two cytokines without providing phenotypic context. Similarly, classical AIM assays typically rely on a narrow set of activation marker pairs, restricting the resolution and breadth of the analysis. In this report, we present a composite AIM assay that expands the antibody detection panel to include up to ten distinct activation marker pairs. This multiplexed approach enhances the sensitivity and specificity of T cell recall detection and allows for a more nuanced characterization of T cell activation states.

A major goal of our work was the development of a cAIM assay for use in cynomolgus monkeys, a model system where immunological tools are often limited due to poor cross-reactivity of available antibodies. We validated our approach using KLH as an archetypal antigen. Notably, we observed a correlation between KLH-specific antibody titers and T cell recall responses, supporting the biological relevance of our assay readouts. To quantify the responses, we first compared the individual AIM marker expression between antigen-stimulated and control (unstimulated) conditions. We then calculated the SI for each marker pair, and we further refined our analysis by introducing two novel metrics: the cAIM-index and the cAIM-score. The cAIM-index is defined as the average SI of all marker pairs matching or exceeding a threshold of 1.2 (based on empirical laboratory experience) and the cAIM-score represents the percentage of marker pairs contributing to the cAIM-index (i.e. displaying SI ≥ 1.2). The introduction of the cAIM-index and cAIM-score in the analysis enhances the confidence in detecting antigen-specific T cell recall responses, even when individual marker signals are modest. It also enables quantitative interpretation in a single numeric format, reducing reliance on multiple graphical comparisons, which is very convenient when many stimulatory conditions or large samples are investigated. By leveraging a broader AIM marker set and the composite metrics, we can capture a more comprehensive picture of the antigen-specific T cell recall response.

Our assays separate CD4^+^ or CD8^+^ total memory responses from those confined to the central memory or effector T cell compartments. This distinction is particularly relevant when comparing responses to different recall antigens or to the same antigen among subjects exposed to various pharmacological agents. Pharmaceuticals may accelerate or decelerate the development of the immune response, thereby compartmentalizing the responding cells into central or effector subpopulations, depending on whether the immune response is in a contraction phase or actively ongoing *in vivo*. This phenomenon was particularly evident in the data reported using OVA (**Figure 3**). OVA is a weakly immunogenic antigen when administered alone, typically requiring the use of adjuvants to elicit strong immune responses. Consequently, the OVA-specific responses displayed by the group of animals receiving OVA alone were distributed evenly between Tcm and Teff compartments or slightly skewed towards the Tcm subpopulations, indicative of an immune contraction process. Conversely, the OVA-specific reactivity in animals receiving OVA in Alhydrogel was predominantly within the Teff cell compartment, suggesting a still ongoing immune process. The included adjuvant extended the duration and increased the magnitude of the immune response by acting both as an antigen depot and an enhancer of innate activation (41).

Historically naïve and memory T cell subpopulations of NHP have been distinguished using CD28 and CD95 antigens (42). This approach, which remain widely employed, served as a workaround when NHP cross-reactive CCR7- CD45R0- and CD45RA-specific antibodies were limited and could not delineate NHP naïve and memory subsets as clearly as the human counterparts (43, 44). On the other hand, the CD28/CD95 differentiation approach also had some weaknesses since some receptors, i.e., CD28, exhibit altered expression pattern following activation or during chronic stimulation and aging (45). While the CD28/CD95 markers separate naïve versus memory T cells reasonably well, by their own do not provide the granularity achieved using contemporary CCR7- and CD45RA-specific antibodies that effectively recognize NHP antigens. Several recent NHP studies have utilized CCR7 and CD45RA to distinguish naïve and memory phenotypes (46, 47). However, optimal approaches, particularly when employing instrumentation that supports large numbers or reagents, might combine these markers with CD27, CD28 and/or CD95 (48). Our report does not advocate for the exclusive use of one T cell subset method. We chose the CCR7/CD45RA approach based on our experience successfully differentiating naïve and memory T cell subsets in both human and NHP (**Supplementary Figures 4–7**), as well as on our desire to enhance the comparability of the cAIM assay across species. Nevertheless, profiting from the large marker repertoire possibilities offered by the mass cytometry platform we performed a comparative cAIM analysis on Tmem, Tcm and Teff subsets segregated using the classical CD28/CD95 gating procedure (42) or the contemporary CCR7/CD45RA strategy selected by us (**Supplementary Figure 8A**). The sole discernible distinction between the two methodologies was a modest diminution in the cAIM responses associated with the EM populations gated using the classical CD28/95 method in respect to our Teff cell population, including both Tem and terminally effector T (Tte) cells (**Supplementary Figure 8B**). We attribute this reduction to the inability of the CD28/CD95 approach to capture all effector T cells, since it frequently overlooks the double-negative CD28/CD95 population (**Supplementary Figure 8A**), which comprises numerous effector T cells (both Tem and Tte, which, nonetheless, are not distinguished by the CD28/CD95 method). Overall, the type of gating strategy CD25/CD95 versus CCR7/CD45RA seems to have only a minor impact on the cAIM outcome. Conversely, this situation could be altered if the antibody clones are modified, necessitating a prior analysis of the potential to differentiate between the various T cell populations before addressing the T cell recall response.

We observed that the informative value of individual analytes varies depending on the nature of the stimulus and the timing of the response. Different memory T cell subsets showed preferential expression of specific AIM markers (as illustrated in **Figure 2F**), underscoring the utility of employing a comprehensive panel of AIM combinations to increase the likelihood of capturing antigen-specific responses. Furthermore, disparities in readouts between CD4^+^ and CD8^+^ T cells, coupled with the potential for bystander activation, emphasizes the limitations of relying solely on a limited set of markers. These findings accentuate the importance of predefining a diverse and representative panel of analytes and consistently applying it, rather than retrospectively focusing on few AIM markers that may not universally provide informative insights on T cell recall responses.

Our work has identified a set of peptides that reliably recapitulate immune responses to AAV9 vectors. These peptides serve as valuable tools for probing AAV9 capsid-specific immunity, which is critical for advancing gene therapy. Although initially designed for human *in vitro* assays, these peptides turned out suitable to investigate AAV9-specific T cell responses in cynomolgus monkeys. Future, studies with individual peptides from the peptide pool may help pinpoint key epitopes recognized by NHP T cells and further refine the assay.

The cAIM assay we developed is compatible with whole blood samples, eliminating the need for isolating specific cell populations. This not only simplifies the workflow but also preserves the physiological context of immune interactions, making it particularly well-suited for preclinical NHP studies and for clinical monitoring in human pediatric populations.

The use of the CyTOF platform provides several important advantages. First, it enables simultaneous acquisition of samples collected at different time points, ensuring consistent, high-dimensional analysis across longitudinal datasets. Second, CyTOF supports large marker panels that enable deep phenotyping across all blood cell populations, providing a comprehensive view of the immune landscape beyond T cells. The workflow is also well suited for CROs, as samples can be processed up to the staining step, frozen, and later acquired in bulk using barcoding strategies. This adds flexibility and reduces batch effects by allowing all samples to be analyzed together. In addition, the procedure can be performed directly on whole blood without lymphocyte isolation, simplifying handling, minimizing processing time, and avoiding the need for preservatives.

In summary, the cAIM assay provides a powerful and scalable platform for monitoring immune responses to biotherapeutics, integrating whole-blood compatibility with high-dimensional analysis and quantitative metrics, such as the cAIM-index and cAIM-score, to support both translational research and clinical applications.

## Materials and methods

### Human samples

Fully anonymized healthy human blood samples were obtained from the Swiss Red Cross Bern with informed consent. The samples were processed into sera or PBMCs. PBMCs were isolated from human blood by standard density-gradient separation procedures using Ficoll-Paque Plus (Amersham) and then stored in liquid nitrogen in a solution of FCS, containing 8% DMSO.

### *In vivo* NHP studies

All procedures with NHP studies presented in this manuscript followed the Animal Welfare Act, the Guide for the Care and Use of Laboratory Animals, the Office of Laboratory Animal Welfare, and fully commensurate with international standards of Good Laboratory Practices.

For the initial assay development (**Figure 1A**), six female, captive-born cynomolgus monkeys (*Macaca fascicularis*, of Mauritius origin) were obtained from Envigo Global Services Inc., Denver, Pennsylvania. At initiation of dosing, monkeys were approximately 24 to 48 months of age and weighed 2 to 4 kg. All six animals were administered SC with 1 mL/animal of KLH (Imject Mariculture Keyhole Limpet Hemocyanin, Thermo Fisher Scientific, Catalog 77600, Lot no. Yl379319) as a solution of 10 mg/mL in sterile water on days 1 and 37. Blood samples were collected within 5 days of animal arrival/transfer during the pre-dose phase, prior to KLH dosing on days 1 and 37 and once on days 4, 8, 15, 22, 29, 43, 51, and 59 to obtain serum for specific antibody determination. Blood samples were also collected on days 15, 37, 53 and 59 for PBMC isolation and cAIM analysis.

For the comparative antigen evaluation (**Figure 3A**), six male, cynomolgus monkeys (*Macaca fascicularis*, of Mauritius origin, sero-negative for AAV9) were obtained from Envigo Global Services Inc., Denver, Pennsylvania. At the initiation of dosing, monkeys were approximately 31 to 43 months of age and weighed 3.5 to 4.2 kg. Animals were assigned to two groups. Animals of group 1 were dosed with KLH (Thermo Fisher Scientific, catalog 77600, Lot: VL316875) and OVA (InvivoGen, catalog vacpova-100, Batch 5822-44-02) via SC injection on days 1 and 29. Animals of group 2 were dosed with OVA - Alhydrogel (InvivoGen, component 1, catalog vacpova-100, Batch 5822-45–01 and component 2 Alhydrogel^®^ adjuvant 2% catalog vac-alu-50, Lot: 5808-45-02) and AAV9-mCherry vectors (VectorBioLabs, Lot 220718–230317 and Lot 220801-230303) via SC injection and slow bolus manual IV injection, respectively, on days 1 and 29. KLH was administered as a suspension in sterile water for injection (Lot: GF3481), at a dose volume of 1 mL/animal and 10 mg/animal. OVA was resuspended in water (InvivoGen, Water-VacciGrade Batch HPV-44-070) to make a 10 mg/mL stock that was further diluted using aseptic techniques in 0.9% sodium chloride for injection, (USP sterile saline, Lots: 1005709 and 9534882) to a final concentration of 5 mg/mL ready to dose at 1 mL/animal. OVA in Alhydrogel was re-suspended in water (InvivoGen, Water-VacciGrade Batch HPV-44-070) to achieve a 10 mg/mL stock, which was sterile filtered using an aseptic technique and further diluted with Alhydrogel to the required concentration of 5 mg/mL, ready to be administered at a dose volume of 1 mL/animal. AAV9-mCherry was diluted to 2.5 x 10^12^ vg/mL in PBS (1X, pH 7.4) and was administered at a dose volume of 1 mL/kg via slow bolus manual IV injection over at least 2 minutes. Blood samples were collected on days 3, 7, 32, 36, and 39 to determine antigen-specific antibody titers in sera and on day 44 for cAIM analysis.

For the comparison of analysis platforms (**Figure 4B**), three female cynomolgus monkeys (*Macaca fascicularis*, of Mauritius origin, sero-negative for AAV9) were obtained from Bioculture Mauritius Ltd., Immokalee, Florida. At initiation of dosing, animals were approximately 32 to 35 months of age, and body weights ranged from 2.6 or 3.4 kg. Animals were administered two single SC injections of 10 mg/animal KLH antigen (Thermo Fisher Scientific, Catalog 77600, Lot YK381966) on days 1 and 28 and one single IV injection of 1 x 10^13^ vp/kg AAV9-empty-capsids (Novartis, Lot PPB42219) on day 1, followed by an IV bolus injection boost with 1 x 10^13^ vg/kg AAV9-mCherry (Novartis, Lot PDS984; PPB-42219) on day 28. All formulations were administered at a volume of 1 mL/animal. Blood samples were collected on days 1 and 28 (prior immunization) and on days 7, 15, 35, and 42 to determine antigen-specific antibody titers in sera and on days 35 and 42 for cAIM analysis on the fluorescent and mass cytometry platform, respectively.

### In-*silico* prediction of AAV9 epitopes

The selection of peptides derived from VP1 protein of AAV9 capsids for T cell epitope mapping was made with the help of the Novartis proprietary algorithm iSHAPE (*in silico* HLA aggretope prediction), which is designed to predict peptide sequences from given proteins with the potential to bind to the peptide-binding groove of HLA-DR molecules. The algorithm was developed using machine learning approaches based on a large library of naturally presented HLA-DR associated peptides, which were eluted from homozygous monocyte-derived human dendritic cells and identified via liquid chromatography-mass spectrometry. The algorithm creates position-specific scoring matrices for eight common HLA-DR alleles and all overlapping 9-mer sequences within a protein of interest are scored accordingly. It uses this information to calculate the likelihood of specific peptide binding to HLA-DR molecules.

In the case of AAV9-VP1, the iSHAPE analysis identified a variety of 9-mers within the top 2 percentile of hits. To minimize the number of peptides, the preselected overlapping 9-mers were consolidated into 27 peptides, ranging from 16 to 24 amino acids in length which covered all predicted potentially immunogenic binding cores sequences.

### Antibody assays

Titers of AAV-specific IgG in human serum samples were determined by ELISA. Briefly, Nunc MaxiSorp 384-well plates (Thermo Scientific) were coated with 1x10^10^ vp/ml in carbonate buffer, 0.1M, pH 9.4 (Thermo Scientific) overnight. Human serum samples were serially diluted at a ratio of 1:3 (starting from a 1:50 dilution) for a total of eleven steps in PBS containing 0.5% BSA. The diluted samples were then incubated on antigen-coated plates for two hours to allow binding. The plates were washed with PBS containing 0.025% Tween-20 (PBST, Sigma-Aldrich), and then incubated with HRP-conjugated goat anti-human IgG (Southern Biotech #2040-05) diluted 1:5000 in PBS 0.5% BSA for 1 hour. Plates were washed with PBST and tetramethylbenzidine (TMB) microwell peroxidase substrate (SeramunBlau Fast, Seramun, Germany) was used to develop the reactions. The binding titer is expressed as the last positive dilution above threshold (threshold is calculated as 2 times the average blanks of the plate).

Antigen-specific IgM and IgG antibodies in sera of NHP were determined using Meso Scale Discovery (MSD) electrochemiluminescence. Blood samples were collected from all animals via the femoral vein. No anti-coagulant was used as serum separator tubes were employed. Blood samples were kept at room temperature and allowed to clot before centrifugation. Samples were centrifuged within 1 hour of collection for approximately 10 minutes in a centrifuge (maintained at 4°C) at 1500 x *g*. The serum was transferred to polypropylene, screw-capped, tubes. Following collection, serum samples were placed on dry ice until stored in a freezer, set to maintain at -60 to -80°C. MSD MULTI-ARRAY 96-well high bind plates (MSD, Catalog L15XB-6) were coated with KLH or OVA at 4 µg/mL or AAV9 at 3 x 10^10^ genome copies/mL (GC/mL). Anti-KLH, anti-OVA and anti-AAV9 titers for both IgM and IgG were determined using a specific electrochemiluminescence (ECL) method employing SULFO-TAG labelled Goat anti-Monkey IgM and Goat anti-Monkey IgG antibodies. ECL was performed using the Meso Scale Discovery System (MSD S600) platform and analyzed within Softmax Pro software to determine sample values.

Anti-KLH, anti-OVA and anti-AAV9 IgM and IgG titers were assessed as a cutpoint titration value. The cutpoint titer is defined as the reciprocal of the interpolated dilution of a sample that crosses a defined cutoff value. To determine the cutoff value, naïve serum from a non-vaccinated subject was repeatedly analyzed in each assay. By establishing a background level with a pool of naïve matrix, a cutpoint was defined. Samples above this cut point were considered positive, while those below were deemed negative. The cutoff value is subsequently determined as the mean of the naïve serum replicates, plus three times the standard deviation of the mean. Standard deviation multipliers are derived from the critical values for a one-tailed t-distribution. This approach avoids setting arbitrary cutoff values and addresses the statistical probability of false positive samples. Serial dilutions of serum samples (for example 1/100, 1/500, 1/2500, 1/12500, 1/62500, etc.) were measured. Samples were diluted until the response values were lower than the established cutoff of the naïve serum control. The cutpoint titer was determined as the point at which the sample’s signal (Y) intersects the cutpoint axis (X), using the slope of the line between the points above and below the cutoff.

### cAIM assay in human samples

Frozen PBMCs from three donors, sero-positive for AAV1, 2, 5, 6, 8 and/or 9, were thawed in complete medium (X-VIVO 15 (Lonza, Catalog 04-418Q) supplemented with 1% (v/v) GlutaMax (Gibco, Catalog 35050-038)), washed and counted. Aliquots of 4x10^6^ viable cells were split into 4 wells of 96-well U-bottom plates (Corning, Catalog 3799) at 1x10^6^ cells/well. Four-replicate sets were left unstimulated (medium containing 0.25 DMSO %) or stimulated with AAV9 capsids at 1x10^11^ vp/mL or with the indicated AAV9-VP1 peptides pools (**Supplementary Table 2**) at 1 µg/mL final, for 20 hours at 37°C, 95% humidity, 8% CO_2_. The next day, all replicates from the same condition were pooled in 5 mL polypropylene tubes (Corning, Catalog 352063) and washed twice with 4 mL Maxpar^®^ Cell Staining Buffer (MCSB) (Standard BioTools, Catalog 201068). Subsequently, cells were blocked with 5 µL Human TruStain FcX (BioLegend, Catalog 422301) and surface barcoded with anti-CD45 antibodies (Clone HI30, Standard BioTools) conjugated to 106Cd, 110Cd, 111Cd, 114Cd and 116Cd, for 15 min at room temperature. Following two washes with MCSB, barcoded cells of the different conditions but same donor were pooled and stained using the Maxpar^®^ Direct Immune Profiling Assay (Standard BioTools, Catalog 201349) combined with the Maxpar^®^ Direct™ T-cell Expansion Panel 3 (Standard BioTools, Catalog 201407) for 30min at room temperature (**Supplementary Table 3**). After staining, the samples were washed twice with MCSB and fixed in 1 mL of a 1.6% Formaldehyde solution (diluted in Maxpar^®^ PBS). Fixed cells were stained in 1 mL of 125 nM Cell-ID Intercalator-Ir (diluted in Fix and Perm Buffer) for 48 h at 4°C and the cell pellets were then transferred to -80°C for long-term storage. Three weeks later, the frozen samples were thawed, washed twice with 2 mL of MSCB followed by two washes with 2 mL of Maxpar^®^ Cell Acquisition Solution Plus (Standard BioTools, Catalog 201244), filtered through a 35 nm cell strainer and split into four tubes to reduce cell density and facilitate acquisition on a CyTOF XT instrument (Standard BioTools) at a rate of 200–500 events/second. Data was randomized, normalized and concatenated through the CyTOF software. The data was further normalized, compensated and de-barcoded using the Bioconductor package CATALYST 1.30.2 in Rstudio (49) applying a separation cut-off of ≥ 0.2. Subsequently, the pre-processed data was analyzed using the OMIQ software from Dotmatics. In detail, data was arcsinh transformed and cleaned using PeacoQC (50). Afterwards data was manually gated to select the main population for Residual, Offset, Width, Event Length, Ir191/DNA1, Ir193/DNA2 and for cells negative for Rh103 (live). These single, living CD45-expressing cells were then gated based on their expression of CD3 and CD4 (CD4^+^ T cells) or CD3 and CD8 (CD8^+^ T cells). CD4^+^ and CD8^+^ T cells were further divided into total memory, central memory and effector compartments based on the expression of CCR7 and CD45RA, i.e., total memory (Tmem) were defined as the complementary population to CCR7^+^ CD45RA^+^, central memory (Tcm) were defined as CCR7^+^ CD45RA^-^, and effector cells (Teff) comprising effector memory (Tem) and CD45RA^+^ terminally differentiated effector (Tte/TEMRA) cells were defined as CCR7^-^ cells independently of whether they were CD45RA^-^ (Tem) or CD45RA^+^ (Tte/TEMRA) cells. (**Supplementary Figure 5**). These cell subsets were then gated for the co-expression of the different AIM combinations and the frequency of AIM expressing cells were exported as percent of parent.

For dimension reduction analysis, data was subsampled to 2 x 10^5^ single, living CD45^+^ events per file and computed using the UMAP algorithm (51) (Neighbors: 15, Minimum Distance: 0.4, Components 2, Metric: Euclidean, Learning Rate: 1, Epochs: 200, Embedding Initialization: spectral; on markers CD123, CD19, CD4, CD8a, CD11c, CD16, CD45RO, CD45RA, CD161, CD27, CD57, CD28, CD38, CD56, TCRgd, CD294, CD197, CD14, CD3, CD20, CD66b, HLA-DR, IgD, CD127) in the OMIQ software (Dotmatics). The output from UMAP was then subjected to the PhenoGraph algorithm (52) (K nearest neighbors: 25, Nearest Neighbors algorithm: Annoy, Distance Metric: Euclidean, Clustering method: Leiden, Leiden Seed: 8111; on markers CD123, CD19, CD4, CD8a, CD11c, CD16, CD45RO, CD45RA, CD161, CD27, CD57, CD28, CD38, CD56, TCRgd, CD294, CD197, CD14, CD3, CD20, CD66b, HLA-DR, IgD, CD127) yielding 32 clusters. Medians of marker expression per cluster were calculated through the Clustered Heatmap algorithm. Clusters were then assigned to different immune cell populations based on the expression of lineage markers and consolidated into 18 cell clusters.

Data was plotted using the OMIQ (Dotmatics) and the GraphPad Prism software (Dotmatics).

### cAIM assays in NHP samples

For the fluorescent cAIM readouts, blood samples (5 mL) were obtained from the femoral vein using Potassium K2 EDTA as an anticoagulant. Blood samples were allowed to equilibrate to ambient temperature over at least 60 minutes following collection from the last animal. PBMCs were isolated through Ficoll^®^ Paque Plus (Cytiva) using standard procedures and remaining red cells were lysed using ACK Lysing buffer (Thermo Fisher Scientific, Catalog A1049201). Viable PBMCs were resuspended in complete medium (X-VIVO 15 (Lonza, Catalog 04-418Q) supplemented with 1% (v/v) GlutaMax (Gibco, Catalog 35050-038)) at 20x10^6^ cells/mL. Aliquots of 100 µL (2.0x10^6^ cells) were plated in 96-well round bottom plates. Wells were topped up to 200 µL with medium alone or medium containing test antigens and cultured for 36–48 h at 37°C, 95% humidity and 8% CO_2_. The test antigens depended on the immunization protocol and corresponding specificity of the cAIM assays. For KLH-specific responses the cultures were stimulated with Imject mcKLH (Thermo Fisher Scientific, Catalog 77600) at a final concentration of 30 μg/mL. When OVA-specific responses were assessed, the cultured cells were exposed to 60 μg/mL OVA (InvivoGen, Catalog vac-pova). AAV9-specific responses were interrogated using a pool (or sub-pools as indicated) of chemically synthesized (Peptides&Elephants) AAV9-VP1 peptides (**Supplementary Table 2**), which were identified using the iSHAPE algorithm as described above. Lyophilized peptides were reconstituted in DMSO and then diluted in culture medium, resulting in a final assay concentration of individual peptides at 5 (**Figure 3**) or 10 (**Figure 5**) μg/mL, and DMSO at 0.125% (**Figure 3**) or 0.25% (**Figure 5**) (v/v). When CD154 (CD40L) was utilized as a reporter AIM, the cell cultures were supplemented with anti-human CD40 blocking antibody (Miltenyi Biotec, Catalog 130-094-133) at a final concentration of 0.5 μg/mL.

After incubation, cells were transferred to 96-well V-bottom plates, washed with Dulbecco’s Phosphate-Buffered Saline (DPBS), stained with LIVE/DEAD™ Fixable Near IR-780 (Life Technologies, Catalog L34992), blocked with Human TruStain FcX (BioLegend, Catalog 422302) in DPBS supplemented with 2% FBS and 2mM EDTA and stained against surface markers for 30–60 minutes at 2-8°C in DPBS supplemented with 2% FBS and 2mM EDTA. The antibody panels included in the cAIM assays used to develop the procedure (6x cAIM, **Figure 1**), are listed in (**Supplementary Table 1**) and the antibody panels used to compare the immunogenicity of the different antigens (10x cAIM, **Figure 3**), or the readout platforms (10x cAIM, **Figure 5**) are listed in (**Supplementary Table 4**). Stained cells were washed twice with Stain Buffer (FBS) (BD Biosciences, Catalog 554656) and acquired in a BD Fortessa X-20 instrument.

Resulting fcs-files were processed using the OMIQ software (Dotmatics). In detail, data was pre-processed using flowAI (53), compensated using AutoSpill (54), scaled and manually gated for single, living CD3^+^CD4^+^ and CD3^+^CD8^+^ Tmem, Tcm and Teff cells based on the expression of CCR7 as shown in **Supplementary Figure 4A** and **Supplementary Figure 6A**. Tmem, Tcm and Teff cells were then gated for expression of the AIM pairs, as exemplified in **Supplementary Figure 4B** and **Supplementary Figure 6B**. The numbers of AIM expressing cells were exported as percent of parent.

For the mass cytometry readouts, blood samples (1.5 mL) were obtained from the femoral vein using collection tubes containing lithium heparin. Unprocessed blood samples from each animal were split into three separate tubes (500 µL in each) and then stimulated by adding 500 µL of either medium containing DMSO (0.25% (v/v) final), KLH (30 µg/mL final), or AAV9-VP1 peptides (10 µg/mL final). The blood-stimulus mixtures were transferred to a 96-well round-bottom plate (Falcon, Catalog 353077) in aliquots of 200 µL per well (i.e., each sample mixture was split into 5 wells). The plates were incubated for 19–24 hours at 37°C, 95% humidity, 8% CO_2_. After incubation, aliquots from the same conditions were harvested and pooled into 15 mL polypropylene tubes and centrifuged for 5 min at 350 x *g*. Most of the supernatant was carefully aspirated, leaving approximately 500 µL rest volume. Subsequently, the samples were blocked using Heparin (Sigma Aldrich, Catalog H3149-10KU) at 300 U/mL final and Human TruStain FcX (BioLegend, Catalog 422302) at 10 µL per sample for 15 min at room temperature. The blocked samples were stained simultaneously with Cell-ID Intercalator-103Rh (Standard BioTools, Catalog 201103A) at a final concentration of 1 µM and a pre-mixed surface antibody solution (**Supplementary Table 5**) was added for 30 min at room temperature. Upon completion of surface staining the erythrocytes were lysed using 500 μL of Cal-Lyse lysing solution (Invitrogen, GAS-010) to each sample, vortexing and incubated for 10 min at room temperature in the dark. The samples were topped up with 6 mL with Maxpar^®^ water (Standard BioTools, Catalog 201069), mixed and incubated for a further 10 min. Once the samples appeared translucent, the cells were washed twice with 3 mL of Maxpar^®^ Cell Staining Buffer (Standard BioTools, Catalog 201068). Afterwards, the cells were resuspended and fixed with 1 mL of a 4% Formaldehyde (diluted Maxpar^®^ PBS) (Thermo Fisher Scientific, Catalog 28908) solution for 10 min at room temperature. Subsequently, the cells were stained with 1 mL of 125 nM Cell-ID Intercalator-Ir solution (Standard BioTools, Catalog 201192A) in Maxpar^®^ Fix and Perm Buffer (Standard BioTools, Catalog 201067) for 60 min in the dark at room temperature. The Iridium-stained cells were then pelleted down through centrifugation and the cell pellets were stored at -80°C. Frozen pellets were shipped overseas, to Novartis labs in Switzerland for data acquisition. In detail, frozen pellets were thawed, washed twice with Maxpar^®^ Cell Staining Buffer followed by two washes with Maxpar^®^ Cell Acquisition Solution Plus. Samples were then filtered through a 35 nm cell strainer and centrifuged. Cell pellets were acquired on a CyTOF XT instrument (Standard BioTools) at a rate of 200–500 events/sec. Data was randomized and normalized through the CyTOF software and then, further compensated using the Bioconductor package CATALYST 1.30.2 in Rstudio (49). Subsequently, the pre-processed data was analyzed using the OMIQ software (Dotmatics). In detail, data was arcsinh scaled and then cleaned using PeacoQC (50). Afterwards data was manually gated, selecting the main population for Residual, Offset, Width, Event Length, Ir191/DNA1, Ir193/DNA2 and for cells negative for Rh103 (live). These single, living CD45-expressing cells were then gated based on their expression of CD3 and CD4 (CD4^+^ T cells) or CD3 and CD8 (CD8^+^ T cells). CD4^+^ and CD8^+^ T cells were further divided into Memory, Central Memory and Effector compartment based on the expression of CCR7 and CD45RA as exemplified in **Supplementary Figure 7A**. These cells were then gated for the co-expression of AIM combinations i.e., CD25/CD69, CD25/CD134, CD25/CD137, CD69/CD134, CD69/CD137 and CD134/CD137 (**Supplementary Figure 7B**). The numbers of AIM expressing cells were exported as percent of parent.

For dimension reduction analysis, data was subsampled to 1.5 x 10^5^ single, living CD45^+^ events expressing either CD3, CD11c, CD14 or C20 (Boolean OR Filter) per file and computed using the UMAP algorithm (51) (Neighbors 15, Minimum Distance 0.4, Components 2, Metric: Euclidean, Learning Rate: 1, Epochs: 200, Random Seed: 8026, Embedding Initialization: spectral; on markers: CD86, CD69, CD45RA, CD14, CD4, CD8, CD11c, CD226, CD25, CD134, CXCR3, CTLA-4, PD-1, CCR7, CD28, CD49b, CCR6, CD95, CD127, TIGIT, CD278, CD3, CD20, CD223, CXCR5, CD137) in OMIQ software (Dotmatics). The output from UMAP was then subjected to the FlowSOM algorithm (55) (xdim: 12, ydim: 12, rlen (# of training iterations): 25, Distance Metric: Euclidean, Run Consensus Metaclustering, Comma-separated k values: 20, Random Seed: 4618) to identify 20 clusters. Medians and abundances of clusters were calculated through the Clustered Heatmap algorithm. Clusters were then assigned to different immune cell populations based on the expression of lineage markers and merged to 9 cell clusters. The edgeR algorithm (56) was used to perform statistical analysis of the observed differences in cluster abundances between control and stimulated conditions (DMSO vs. AAV9, DMSO vs. KLH). Data was plotted using the OMIQ (Dotmatics) and the GraphPad Prism software (Dotmatics) (**Supplementary Figure 7B**; **Figure 6**).

All antibodies utilized in the NHP primate experiments (both fluorescent and mass cytometry) were tested for cross-reactivity to cynomolgus monkeys (*Macaca fascicularis*), since some clones labelled as NHP cross-reactive might bind to antigens in rhesus monkeys (*Macaca mulatta*) but not necessarily to the cynomolgus species. The specific dilutions for each antibody are provided in the supplementary tables detailing the various antibody panels.

### cAIM analysis

To calculate the different SI the data was first evaluated to determine the minimal expression value of each AIM marker pair that was >0 in the CD4^+^ and CD8^+^ T cells. Such value was then added to all observations within the given activation marker combination in the corresponding CD4^+^ or CD8^+^ T cell populations using an R script in Rstudio. The zero-normalized values were used as input to calculate fold-changes of the antigen stimulation over the corresponding medium control (SI), which now will not have the possibility to contain a zero denominator. Both the individual expression values as well as the SI were plotted using the GraphPad Prism (Dotmatics) software. As further refinement we calculated a cAIM-index by averaging all SI values ≥1.2 for a given test sample (e.g. across 6–10 AIM pairs within each T cell subpopulation). The threshold value of 1.2 was selected based on laboratory experience using a wide variety of samples from different donors. The 99.99th of our laboratory control AIM responses (plus two standard deviation) consistently remains below 0.4. Consequently, to quantify the validity of the test responses we defined a Sensitivity Criterion (SensCrit) for each AIM pair as:


$$SensCrit=\frac{Expression\;value\;of\;antigen\;stimulated\;sample}{Expression\;value\;of\;control\;sample+Expression\;value\;of\;antigen\;stimulated\;sample}$$

Using this formula, we considered AIM expression responses as true when their SensCrit ≥0.545, which corresponds to an SI of ≥1.2, and neglects all others. Ideally, all AIM pairs in each assay should meet the ≥1.2 threshold criterion. However, some tests will fail (e.g., when the antigen-specific response is too weak or inexistent or when some markers are not expressed on a particular T cell subpopulation) and then, it is important to determine the consistency of the AIM expression. To this end, a cAIM-score, defined as the percentage of AIM pairs that meet the SI ≥1.2 criterion is very useful. In practice a cAIM-score of 100% is desirable for a T cell recall response, but this might not occur always. Hence, comparing the AIM scores across different stimuli or assay conditions, or examining the cAIM-index of samples with identical cAIM-scores, can provide additional insights into the magnitude and quality of the responses being investigated. Furthermore, for comparison of responses among different populations (e.g., differing in treatment or kinetics) the cAIM-index and cAIM-scores of a group of animals could be consolidated into group cAIM-index and group cAIM-score by averaging the corresponding cAIM metrics of the individual subjects.

The antibodies used in the human and NHP fluorescent and mass cytometry experiments directed to CCR7 and CD45RA antigens permit the discrimination of T cells subsets on at least naïve (CCR7^+^CD45RA^+^), central memory (CCR7^+^CD45RA^-^), effector memory (CCR7^-^CD45RA^-^) and terminally differentiated effector (CCR7^-^CD45RA^+^) subsets. To simplify our analysis, we restricted our gating strategy to central memory T cells (Tcm) defined as CCR7^+^CD45RA^-^ cells; to effector T cells (Teff) comprising CCR7^-^CD45RA^-^ and/or CCR7^-^CD45RA^+^ cells; and total memory T cells (Tmem) defined as the complementary population of CCR7^+^CD45RA^+^. Additional granularity is possible, particularly in the mass cytometry experiments but these subdivisions were not on the scope of this study.

## Data Availability

The datasets presented in this article are not readily available because the article does not contain deep genomic, proteomic or sequencing data. Requests to access the datasets should be directed to jan.schmidt@novartis.com.
