# Supplementary figures and images for: Assessment of antigen-specific T cell recall responses in non-human primates using a composite AIM assay
Source: Front Immunol. 2025 Oct 24;16:1661480. doi: 10.3389/fimmu.2025.1661480 (PMC12592150; doi:10.3389/fimmu.2025.1661480)

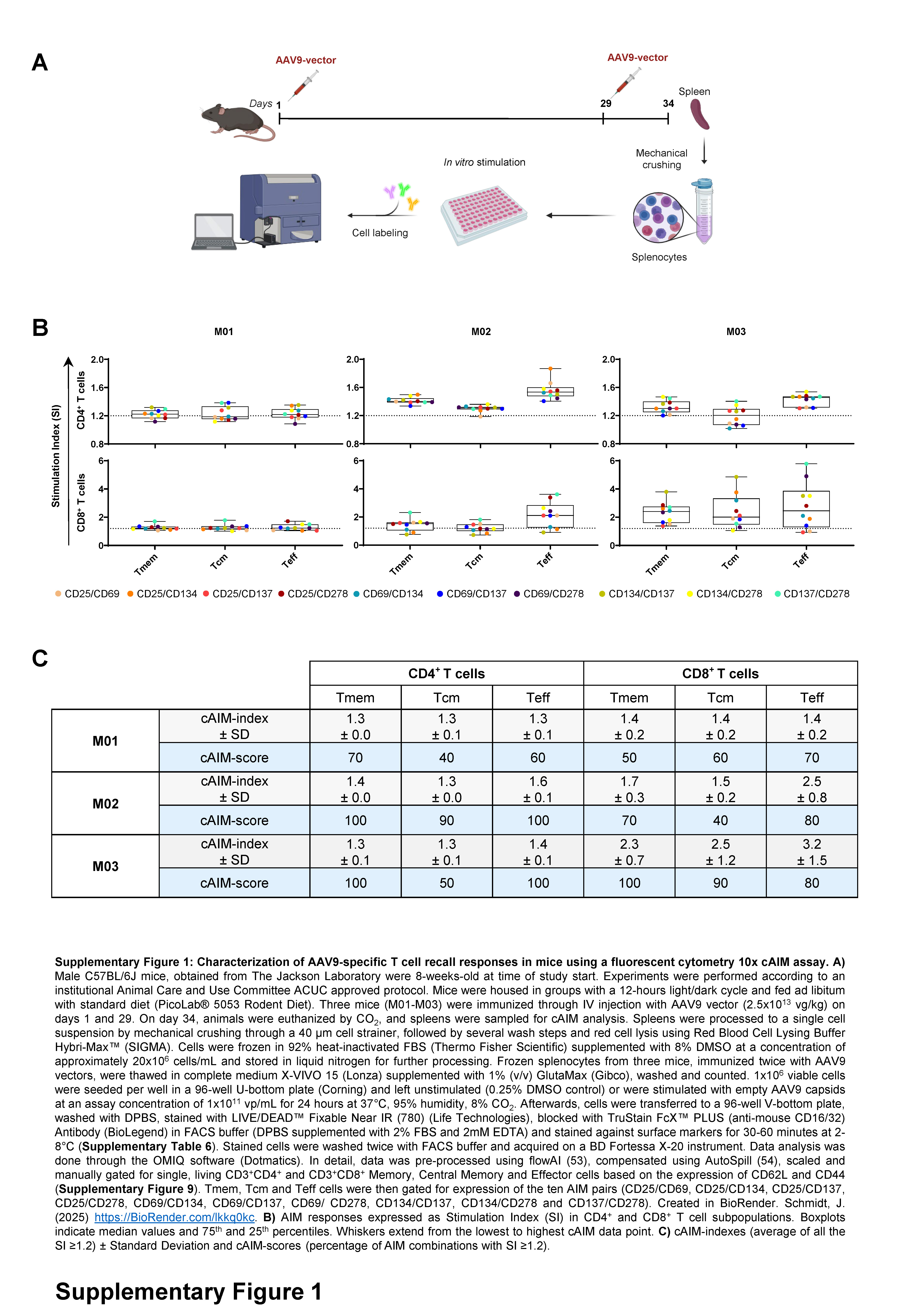

Supplement: Supplementary file 1 [file Image1.jpeg]

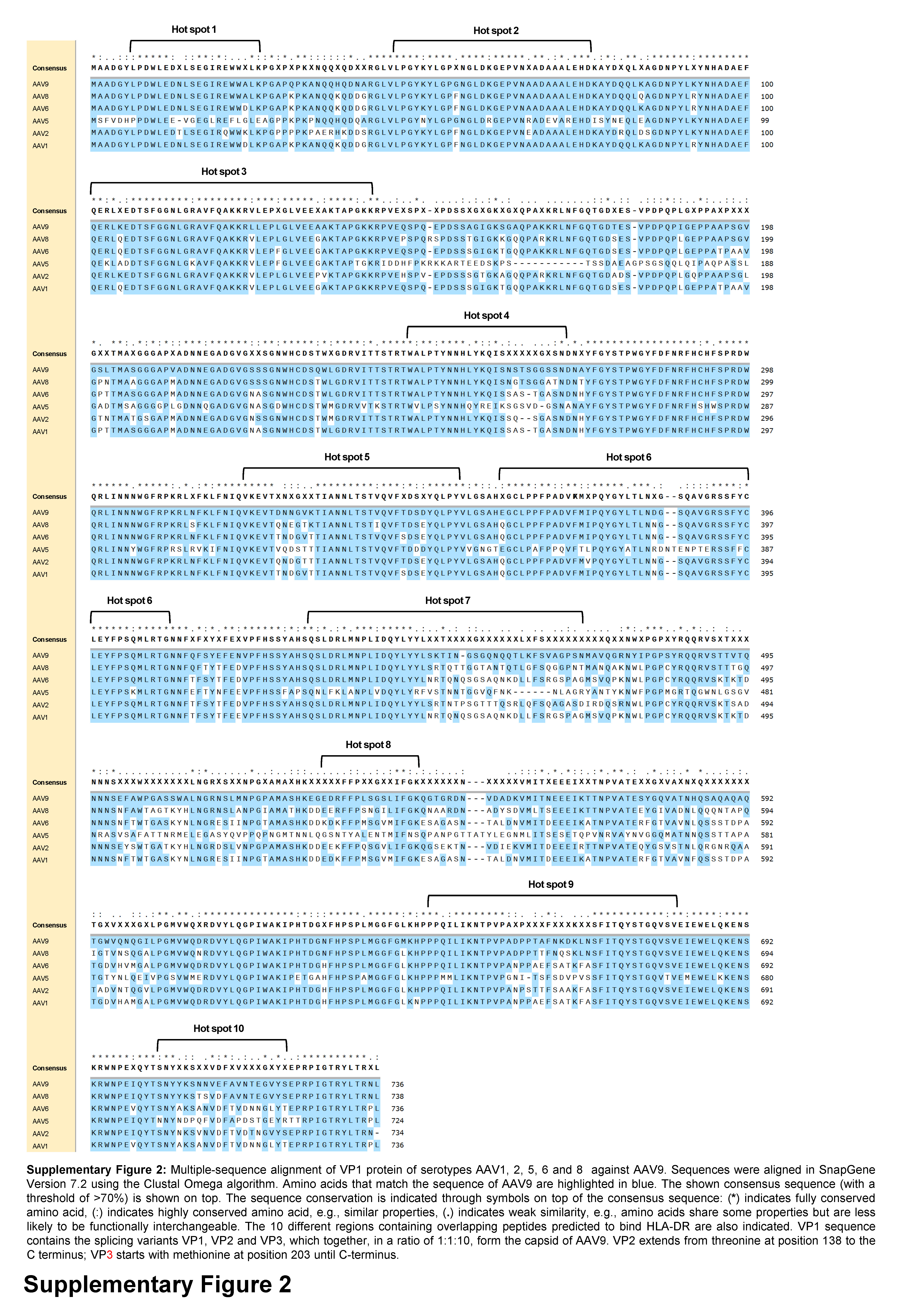

Supplement: Supplementary file 2 [file Image2.jpeg]

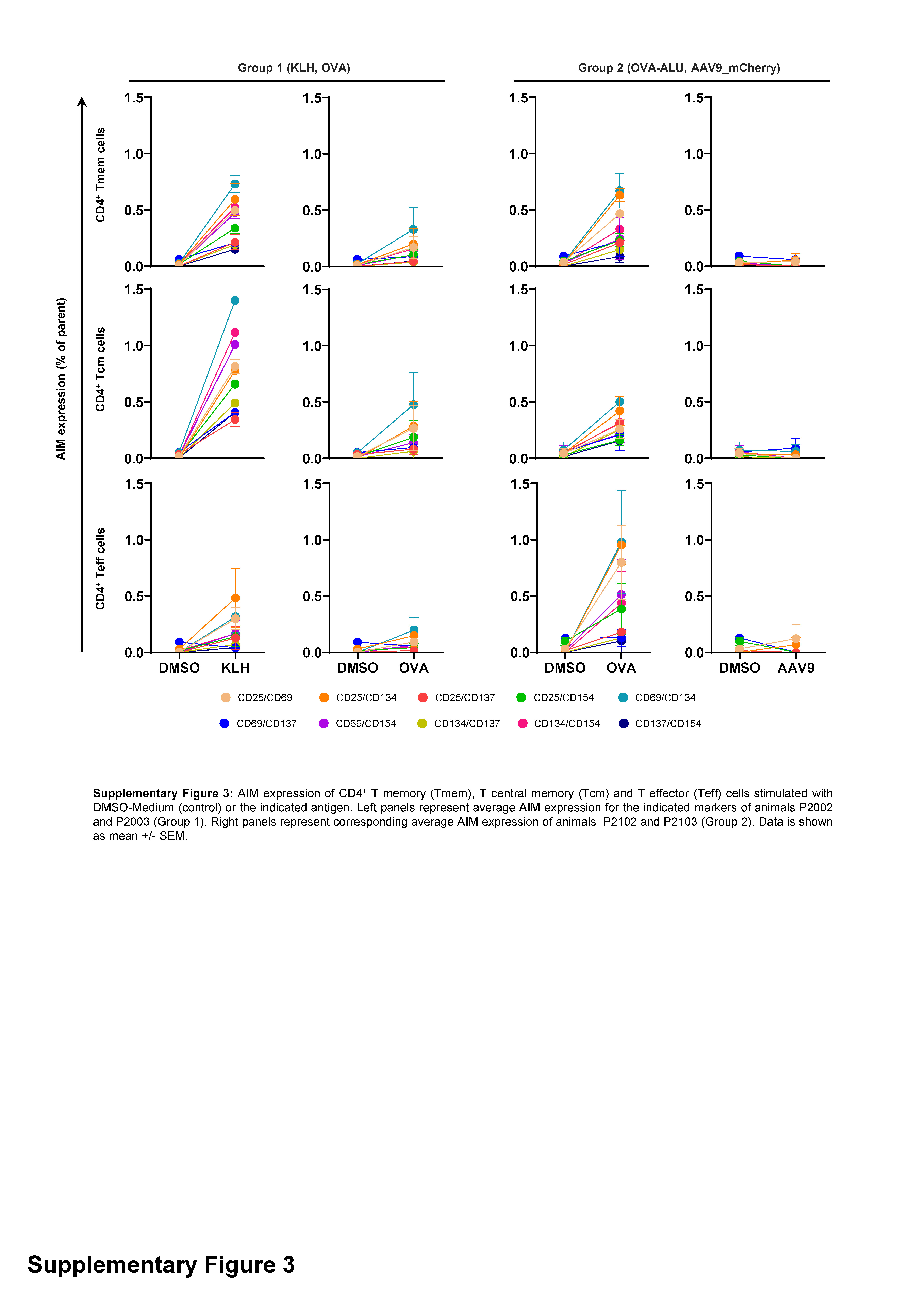

Supplement: Supplementary file 3 [file Image3.jpeg]

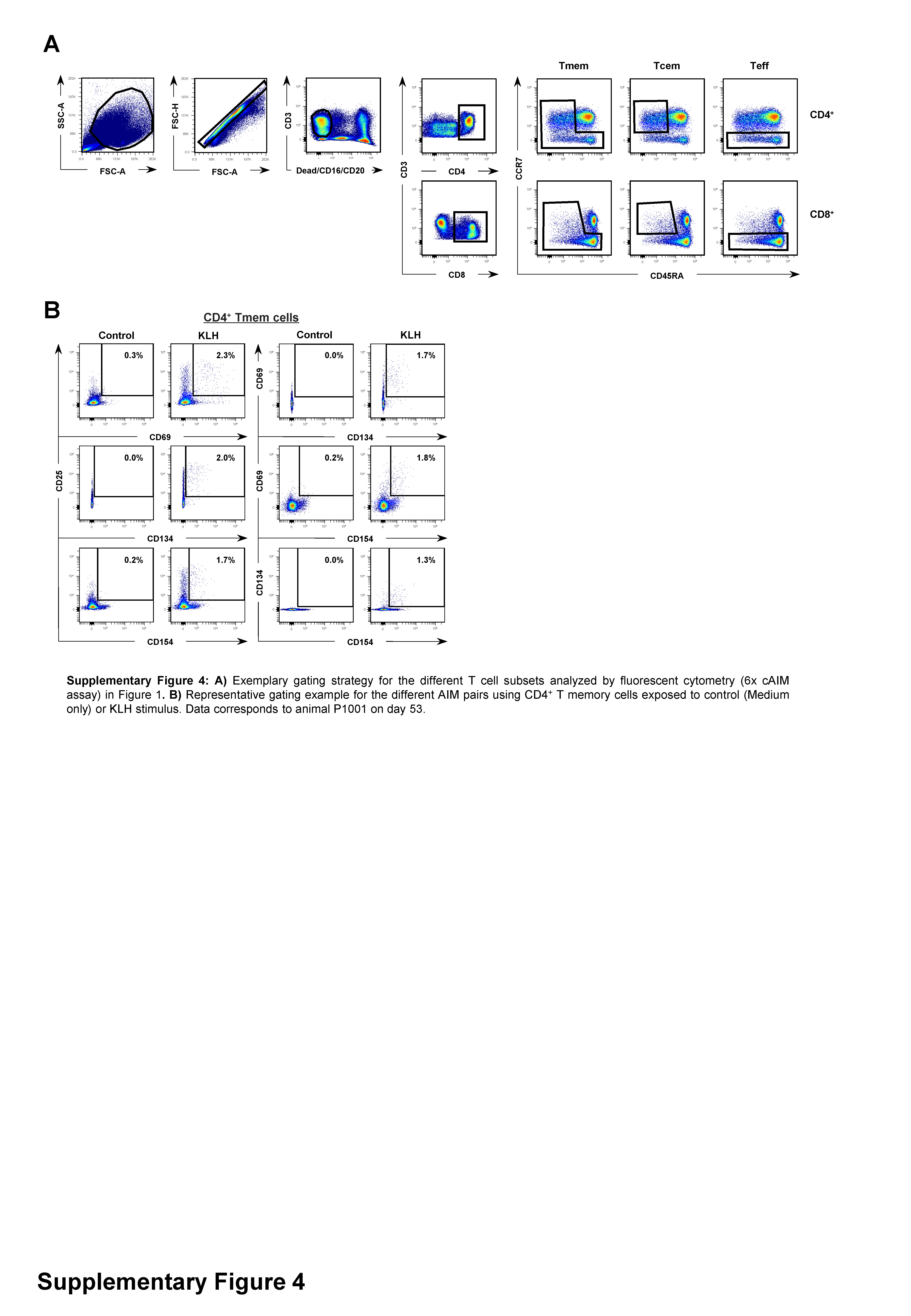

Supplement: Supplementary file 4 [file Image4.jpeg]

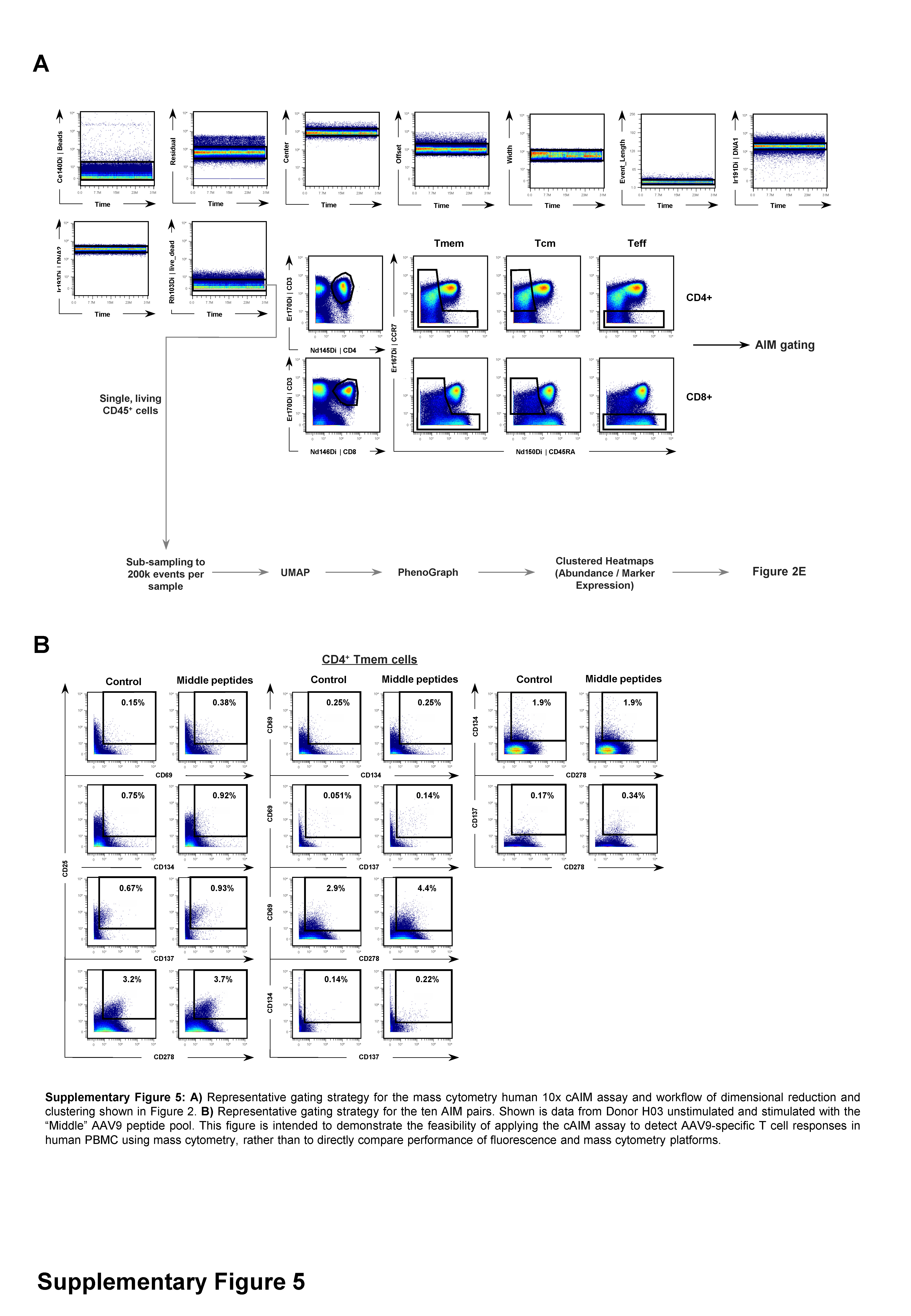

Supplement: Supplementary file 5 [file Image5.jpeg]

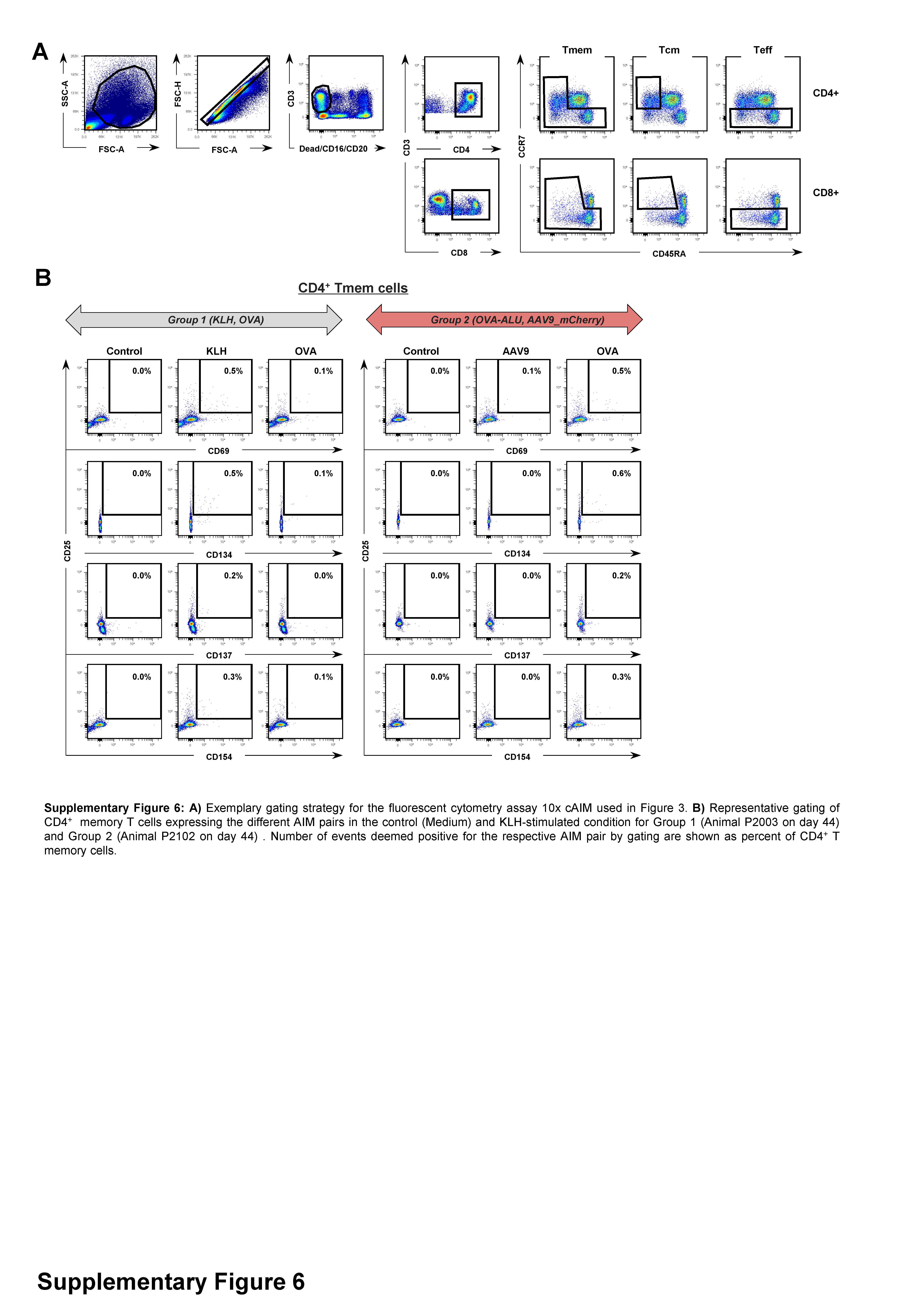

Supplement: Supplementary file 6 [file Image6.jpeg]

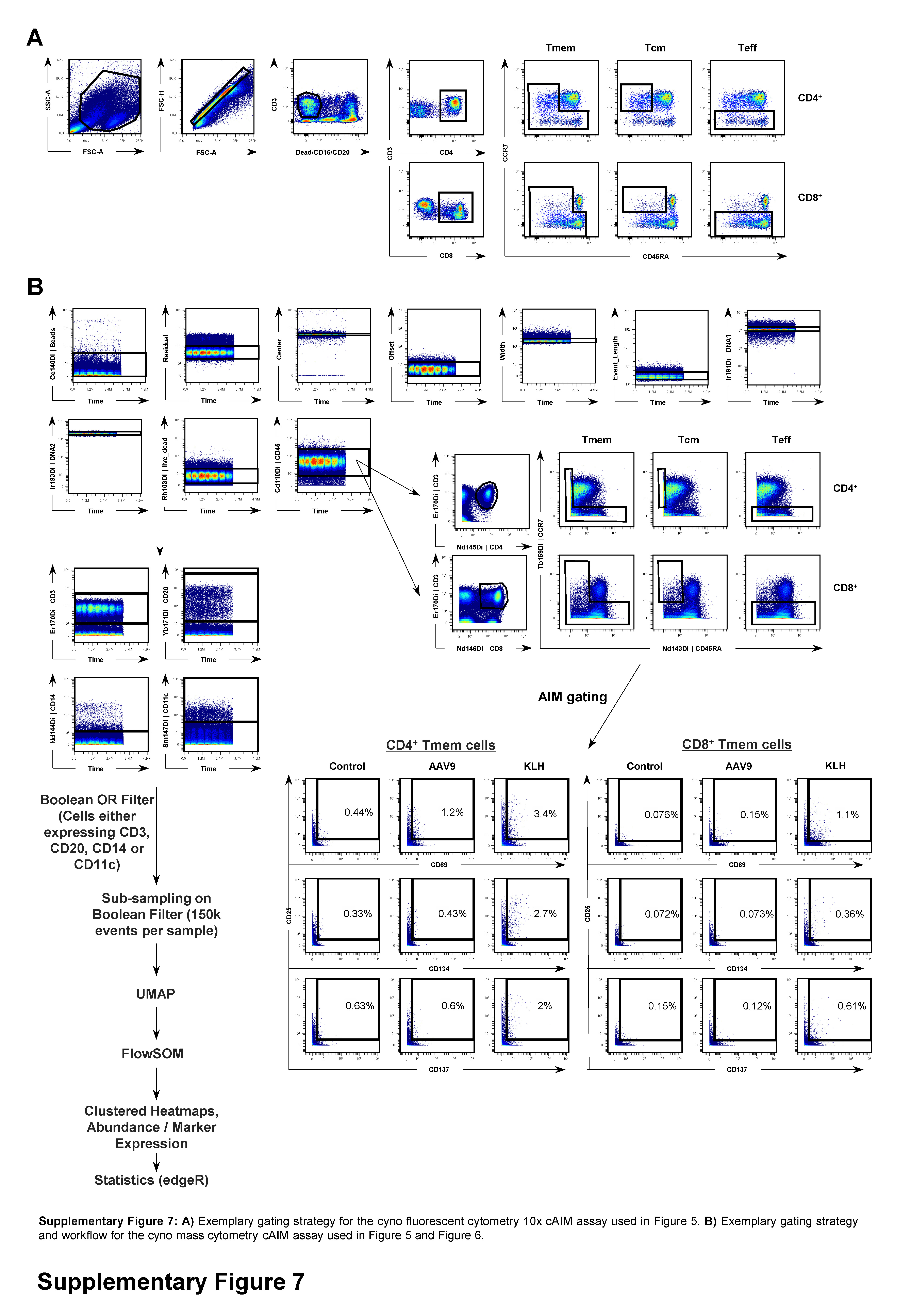

Supplement: Supplementary file 7 [file Image7.jpeg]

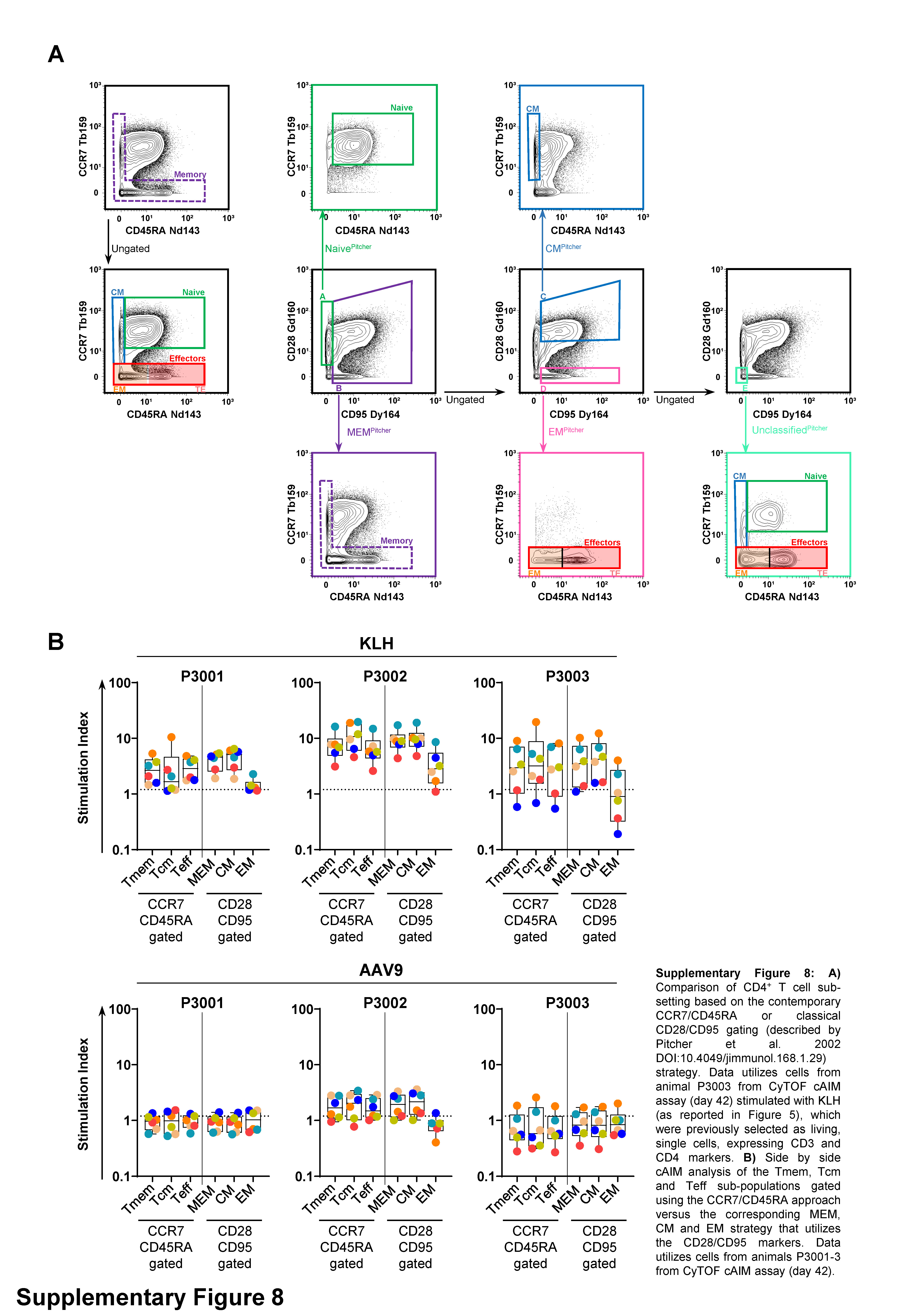

Supplement: Supplementary file 8 [file Image8.jpeg]

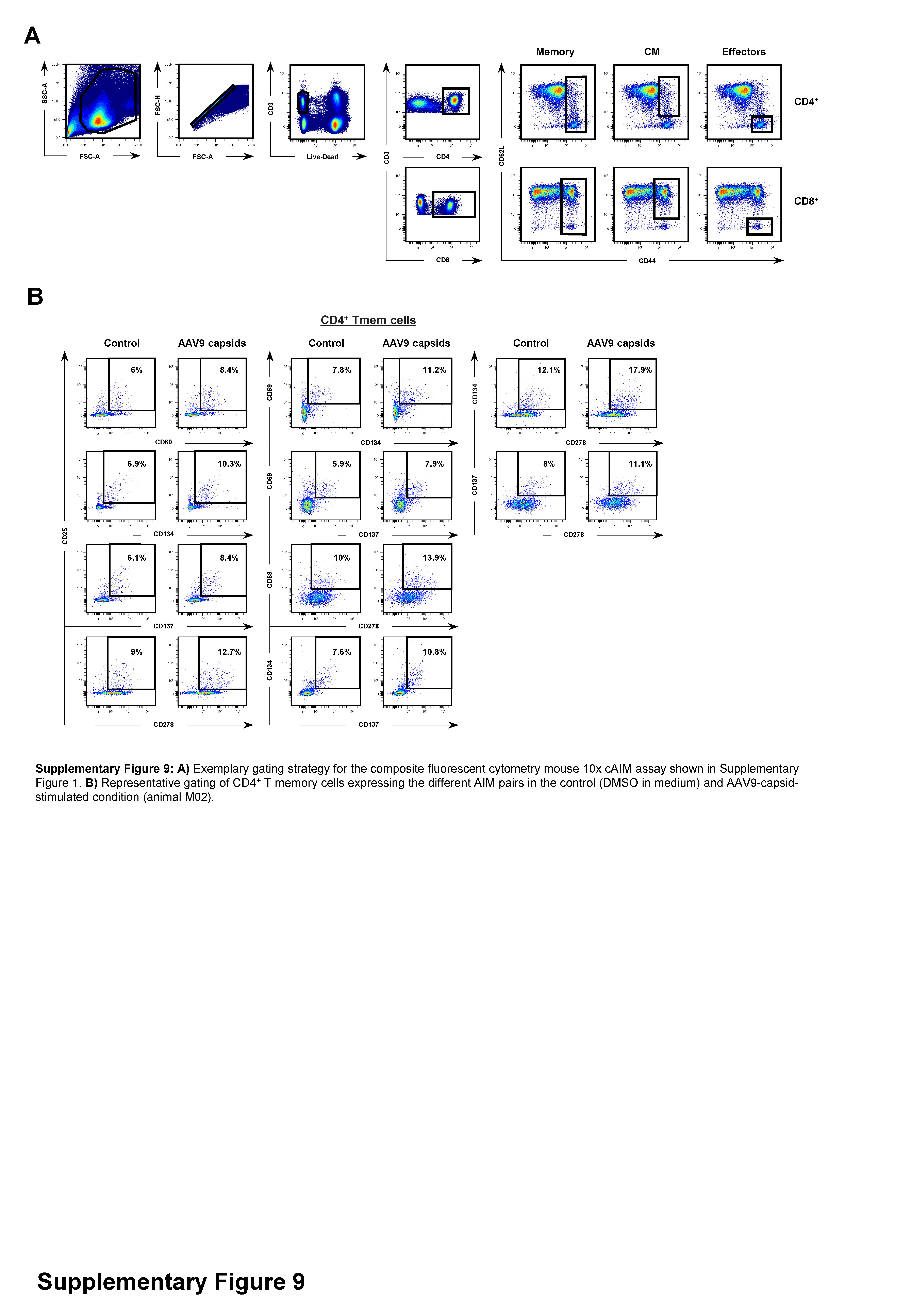

Supplement: Supplementary file 9 [file Image9.jpeg]
