# Supplementary material for: Assessment of antigen-specific T cell recall responses in non-human primates using a composite AIM assay
Source: Front Immunol. 2025 Oct 24;16:1661480. doi: 10.3389/fimmu.2025.1661480 (PMC12592150; doi:10.3389/fimmu.2025.1661480)
Supplement: Supplementary file 10 [file Table1.docx]

**Supplementary Table 1**

| **Target** | **Clone** | **Conjugate** | **Vendor** | **Cat. Number** | **Dilution** |
| --- | --- | --- | --- | --- | --- |
| CD3 | SP34-2 | BUV395 | BD Biosciences | 564117 | 1:100 |
| CD4 | L200 | BV711 | BD Biosciences | 563913 | 1:50 |
| CD8 | RPA-T8 | BV510 | BioLegend | 301048 | 1:50 |
| CD25 | BC96 | FITC | BioLegend | 302604 | 1:100 |
| CD45RA | 5H9 | BB700 | BD Biosciences | 742249 | 1:100 |
| CD69 | FN50 | BV605 | BioLegend | 310938 | 1:100 |
| CD127 | HIL-7R-M21 | BV421 | BD Biosciences | 562436 | 1:50 |
| CD134 | L106 | RY586 | BD Biosciences | 753310 | 1:100 |
| CD154 | 5C8 | APC | Miltenyi | 130-113-603 | 1:100 |
| CD197 | G043H7 | PE-Cy7 | BioLegend | 353226 | 1:50 |
| CD20 | 2H7 | APC-Fire750 | BioLegend | 302358 | 1:200 |
| CD16 | 3G8 | APC-Fire750 | BioLegend | 302060 | 1:100 |

**Supplementary Table 2**

| **Peptide number** | **Amino Acids** | **N-Term** | **Sequence** | **C-Term** | **Length** | **Hot spot** | **Peptide Pool** | |
| --- | --- | --- | --- | --- | --- | --- | --- | --- |
| 1 | 007-026 | H | LPDWLEDNLSEGIREWWALK | NH2 | 20 | 1 | N-terminal (007-270aa) | **All** |
| 2 | 047-061 | H | LPGYKYLGPGNGLDK | NH2 | 15 | 2 |  |  |
| 3 | 062-076 | H | GEPVNAADAAALEHD | NH2 | 15 |  |  |  |
| 4 | 101-122 | H | QERLKEDTSFGGNLGRAVFQAK | NH2 | 22 | 3 |  |  |
| 5 | 114-133 | H | LGRAVFQAKKRLLEPLGLVE | NH2 | 20 |  |  |  |
| 6 | 120-139 | H | QAKKRLLEPLGLVEEAAKTA | NH2 | 20 |  |  |  |
| 7 | 124-143 | H | RLLEPLGLVEEAAKTAPGKK | NH2 | 20 |  |  |  |
| 8 | 247-265 | H | WALPTYNNHLYKQISNSTS | NH2 | 19 | 4 |  |  |
| 9 | 253-270 | H | NNHLYKQISNSTSGGSSN | NH2 | 18 |  |  |  |
| 10 | 322-341 | H | VKEVTDNNGVKTIANNLTST | NH2 | 20 | 5 | Middle (322-456aa) |  |
| 11 | 328-346 | H | NNGVKTIANNLTSTVQVFT | NH2 | 19 |  |  |  |
| 12 | 339-353 | H | TSTVQVFTDSDYQLP | NH2 | 15 |  |  |  |
| 13 | 361-376 | H | EGCLPPFPADVFMIPQ | NH2 | 16 | 6 |  |  |
| 14 | 369-388 | H | ADVFMIPQYGYLTLNDGSQA | NH2 | 20 |  |  |  |
| 15 | 375-392 | H | PQYGYLTLNDGSQAVGRS | NH2 | 18 |  |  |  |
| 16 | 391-408 | H | RSSFYCLEYFPSQMLRTG | NH2 | 18 |  |  |  |
| 17 | 430-449 | H | QSLDRLMNPLIDQYLYYLSK | NH2 | 20 | 7 |  |  |
| 18 | 437-456 | H | NPLIDQYLYYLSKTINGSGQ | NH2 | 20 |  |  |  |
| 19 | 456-470 | H | QNQQTLKFSVAGPSN | NH2 | 15 | 7 | C-terminal (456-722aa) |  |
| 20 | 531-545 | H | EDRFFPLSGSLIFGK | NH2 | 15 | 8 |  |  |
| 21 | 644-663 | H | PPQILIKNTPVPADPPTAFN | NH2 | 20 | 9 |  |  |
| 22 | 650-669 | H | KNTPVPADPPTAFNKDKLNS | NH2 | 20 |  |  |  |
| 23 | 655-674 | H | PADPPTAFNKDKLNSFITQY | NH2 | 20 |  |  |  |
| 24 | 661-680 | H | AFNKDKLNSFITQYSTGQVS | NH2 | 20 |  |  |  |
| 25 | 666-681 | H | KLNSFITQYSTGQVSV | NH2 | 16 |  |  |  |
| 26 | 703-716 | H | SNYYKSNNVEFAVN | NH2 | 14 | 10 |  |  |
| 27 | 708-722 | H | SNNVEFAVNTEGVYS | NH2 | 15 |  |  |  |

**Supplementary Table 3**

| **Maxpar® Direct™ Immune Profiling Assay™** | | | |
| --- | --- | --- | --- |
| **Target** | **Clone** | **Metal** | **Dilution** |
| CD45 | HI30 | 89Y | ~6 Mio. living PBMCs per lyophilized antibody  pellet. |
| CD196/CCR6 | G034E3 | 141Pr |  |
| CD123 | 6H6 | 143Nd |  |
| CD19 | HIB19 | 144Nd |  |
| CD4 | RPA-T4 | 145Nd |  |
| CD8a | RPA-T8 | 146Nd |  |
| CD11c | Bu15 | 147Sm |  |
| CD16 | 3G8 | 148Nd |  |
| CD45RO | UCHL1 | 149Sm |  |
| CD45RA | HI100 | 150Nd |  |
| CD161 | HP-3G10 | 151Eu |  |
| CD194/CCR4 | L291H4 | 152Sm |  |
| CD25 | BC96 | 153Eu |  |
| CD27 | O323 | 154Sm |  |
| CD57 | HNK-1 | 155Gd |  |
| CD183/CXCR3 | G025H7 | 156Gd |  |
| CD185/CXCR5 | J252D4 | 158Gd |  |
| CD28 | CD28.2 | 160Gd |  |
| CD38 | HB-7 | 161Dy |  |
| CD56/NCAM | NCAM16.2 | 163Dy |  |
| TCRgd | B1 | 164Dy |  |
| CD294 | BM16 | 166Er |  |
| CD197/CCR7 | G043H7 | 167Er |  |
| CD14 | 63D3 | 168Er |  |
| CD3 | UCHT1 | 170Er |  |
| CD20 | 2H7 | 171Yb |  |
| CD66b | G10F5 | 172Yb |  |
| HLA-DR | LN3 | 173Yb |  |
| IgD | IA6-2 | 174Yb |  |
| CD127 | A019D5 | 176Yb |  |
| **Maxpar® Direct™ T Cell Expansion Panel 3** | | | |
| **Target** | **Clone** | **Metal** | **Dilution** |
| CD134/OX40 | ACT35 | 142Nd | 1:100 |
| TIGIT | MBSA43 | 159Tb | 1:100 |
| CD69 | FN50 | 162Dy | 1:300 |
| CD279/PD-1 | EH12.2H7 | 165Ho | 1:100 |
| CD366/Tim-3 | F38-2E2 | 169Tm | 1:100 |
| CD278/ICOS | C398.4A | 175Lu | 1:100 |
| CD137/4-1BB | 4B4-1 | 209Bi | 1:150 |

**Supplementary Table 4**

| **Target** | **Clone** | **Conjugate** | **Vendor** | **Cat. Number** | **Dilution** |
| --- | --- | --- | --- | --- | --- |
| CD3 | SP34-2 | BUV395 | BD Biosciences | 564117 | 1:100 |
| CD4 | L200 | BV711 | BD Biosciences | 563913 | 1:50 |
| CD8 | RPA-T8 | BV510 | BioLegend | 301048 | 1:50 |
| CD25 | BC96 | FITC | BioLegend | 302604 | 1:100 |
| CD45RA | 5H9 | BB700 | BD Biosciences | 742249 | 1:100 |
| CD69 | FN50 | BV605 | BioLegend | 310938 | 1:100 |
| CD137 | 4B4 | Super Bright 436 | Invitrogen | 62-1379-42 | 1:100 |
| CD134 | L106 | RY586 | BD Biosciences | 753310 | 1:100 |
| CD154 | 5C8 | APC | Miltenyi | 130-113-603 | 1:100 |
| CD197 | G043H7 | PE-Cy7 | BioLegend | 353226 | 1:50 |
| CD20 | 2H7 | APC-Fire750 | BioLegend | 302358 | 1:200 |
| CD16 | 3G8 | APC-Fire750 | BioLegend | 302060 | 1:100 |

**Supplementary Table 5**

| **Target** | **Clone** | **Metal** | **Vendor** | **Cat. Number** | **In-house labeling** | **Dilution** |
| --- | --- | --- | --- | --- | --- | --- |
| CD86 | IT2.2 | 106 Cd | Invitrogen | 14-0869-82 | Yes | 1:500 |
| CD45 | D058-1283 | 110 Cd | BD Pharmingen | 552566 | Yes | 1:500 |
| CD69 | FN50 | 113 Cd | StandardBioTools | 3113002B | No | 1:500 |
| CD45RA | 5H9 | 143 Nd | StandardBioTools | 3999999-2 | No | 1:500 |
| CD14 | M5E2 | 144 Nd | BioLegend | 301843 | Yes | 1:500 |
| CD4 | "OKT4" | 145 Nd | StandardBioTools | 92J034145 | No | 1:500 |
| CD8 | RPA-T8 | 146 Nd | StandardBioTools | 3146001B | No | 1:500 |
| CD11c | 3.9 | 147 Sm | StandardBioTools | 92J038147 | No | 1:500 |
| CD226 | 11A8 | 148 Nd | BioLegend | 338302 | Yes | 1:250 |
| CD25 | 2A3 | 149 Sm | StandardBioTools | 3149010B | No | 1:500 |
| CD134 | L106 | 150 Nd | StandardBioTools | 3999999-2 | No | 1:500 |
| CD183(CXCR3) | G025H7 | 152 Sm | BioLegend | 353733 | Yes | 1:250 |
| CD40 | 5C3 | 155 Gd | BD Pharmingen | 555587 | Yes | 1:1000 |
| CD152(CTLA-4) | BNI3.1 | 156 Gd | BD Pharmingen | 555851 | Yes | 1:500 |
| CD279 | EH12.2H7 | 158 Gd | BioLegend | 329941 | Yes | 1:1000 |
| CD197 | G043H7 | 159 Tb | StandardBioTools | 3159003A | No | 1:500 |
| CD28 | CD28.2 | 160 Gd | StandardBioTools | 3160003B | No | 1:500 |
| CD49b | AK7 | 161 Dy | BD Pharmingen | 555497 | Yes | 1:500 |
| CD196 (CCR6) | G034E3 | 163 Dy | BioLegend | 353427 | Yes | 1:250 |
| CD95 | DX2 | 164 Dy | StandardBioTools | 3164008B | No | 1:1000 |
| CD127 | A019D5 | 165 Ho | StandardBioTools | 3165008B | No | 1:250 |
| TIGIT | A15153G | 167 Er | BioLegend | 372702 | Yes | 1:250 |
| CD278/ICOS | C398.4A | 169 Tm | StandardBioTools | 3169030B | No | 1:500 |
| CD3 | SP34-2 | 170 Er | StandardBioTools | 3170007B | No | 1:500 |
| CD20 | 2H7 | 171 Yb | StandardBioTools | 3171012B | No | 1:500 |
| CD223 (LAG-3) | REA351 | 174 Yb | Miltenyi Biotec | 130-124-529 | Yes | 1:250 |
| CD185(CXCR5) | J252D4 | 176 Yb | BioLegend | 356902 | Yes | 1:250 |
| CD137 | 4B4-1 | 209 Bi | StandardBioTools | 3209015B | No | 1:250 |

**Supplementary Table 6**

| **Target** | **Clone** | **Conjugate** | **Vendor** | **Cat. Number** | **Dilution** |
| --- | --- | --- | --- | --- | --- |
| CD3 | 145-2C11 | BUV395 | BD Biosciences | 563565 | 1:100 |
| CD4 | GK1.5 | BV711 | Biolegend | 100447 | 1:50 |
| CD8 | 53-6.7 | eFluor506 | Thermo Fisher Scientific | 69-0081-82 | 1:100 |
| CD25 | 7D4 | FITC | BD Biosciences | 553072 | 1:100 |
| CD44 | IM7 | BB700 | BD Biosciences | 566506 | 1:100 |
| CD62L | MEL-14 | PE-Cy7 | Thermo Fisher Scientific | 25-0621-82 | 1:50 |
| CD69 | H1.2F3 | BV605 | Biolegend | 104530 | 1:100 |
| CD134 | OX-86 | PE-Dazzle594 | Biolegend | 119418 | 1:100 |
| CD137 | 17B5 | eFluor450 | ThermoFisher Scientific | 48-1371-82 | 1:100 |
| CD278 | 15F9 | APC | Biolegend | 107712 | 1:100 |
